# Keratinocyte differentiation promotes ER stress-dependent lysosome biogenesis

**DOI:** 10.1038/s41419-019-1478-4

**Published:** 2019-03-19

**Authors:** Sarmistha Mahanty, Shruthi Shirur Dakappa, Rezwan Shariff, Saloni Patel, Mruthyunjaya Mathapathi Swamy, Amitabha Majumdar, Subba Rao Gangi Setty

**Affiliations:** 1 Department of Microbiology and Cell Biology, Indian Institute of Science, Bangalore, 560012 India; 2 Unilever R&D, Bangalore, 560066 India

**Keywords:** Calcium and vitamin D, Lysosomes, Calcium signalling

## Abstract

Keratinocytes maintain epidermal integrity through cellular differentiation. This process enhances intraorganelle digestion in keratinocytes to sustain nutritional and calcium-ionic stresses observed in upper skin layers. However, the molecular mechanisms governing keratinocyte differentiation and concomitant increase in lysosomal function is poorly understood. Here, by using primary neonatal human epidermal keratinocytes, we identified the molecular link between signaling pathways and cellular differentiation/lysosome biogenesis. Incubation of keratinocytes with CaCl_2_ induces differentiation with increased cell size and early differentiation markers. Further, differentiated keratinocytes display enhanced lysosome biogenesis generated through ATF6-dependent ER stress signaling, but independent of mTOR-MiT/TFE pathway. In contrast, chemical inhibition of mTORC1 accelerates calcium-induced keratinocyte differentiation, suggesting that activation of autophagy promotes the differentiation process. Moreover, differentiation of keratinocytes results in lysosome dispersion and Golgi fragmentation, and the peripheral lysosomes showed colocalization with Golgi-tethering proteins, suggesting that these organelles possibly derived from Golgi. In line, inhibition of Golgi function, but not the depletion of Golgi-tethers or altered lysosomal acidity, abolishes keratinocyte differentiation and lysosome biogenesis. Thus, ER stress regulates lysosome biogenesis and keratinocyte differentiation to maintain epidermal homeostasis.

## Abstract (web summary)

Lysosomes are the key digestive organelles of differentiated keratinocytes in the epidermis. Mahanty et al. show that ER stress but not mTOR-MiT/TFE factors promotes lysosome biogenesis during keratinocyte differentiation, which is critical for epidermal homeostasis.

## Introduction

Human epidermis is majorly composed of proliferative and differentiated keratinocytes organized into four distinct layers^1,2^. These sublayers are characterized by specific gene expression and predicted to be formed due to the pre-existing calcium gradient in the layers^3–6^. Proliferative keratinocytes of stratum basale constantly undergo differentiation towards the upper layers and form stratum corneum, and thus maintains epidermal homeostasis. Additionally, terminally differentiated keratinocytes lose their intracellular organelles including nuclei by increasing macroautophagy^7,8^. However, the mechanism of calcium influence on cellular differentiation and its link to organelle homeostasis is poorly understood.

Extracellular high calcium is known to induce keratinocyte differentiation by elevating intracellular calcium through PI3K (phosphoinositide 3-kinase)-dependent IP3R (inositol triphosphate receptor) activation^9,10^. Further, PI3K in concert with mTORC (mammalian target of rapamycin complex) 1, AMPK (AMP-activated protein kinase), and AKT (protein kinase B) has been shown to activate transcriptional response in response to stress including high calcium^11–15^ and sometimes cells undergo differentiation^9,16,17^. Similarly, mTORC1-dependent MiT/TFE transcription factor (TF) TFEB has been shown to regulate the lysosome biogenesis and autophagy in many cell types^18–22^ and also implicated in osteoblast differentiation^23^. Moreover, autophagy is required for epidermal differentiation in vivo and during calcium-induced keratinocytes differentiation in vitro^7,8,24^. Lysosomes are known to play a key role in regulating autophagy including its turnover/flux^18,19,21^. But, whether the increased autophagy also requires enhanced lysosome biogenesis during keratinocyte differentiation has not been addressed. Consistent to this hypothesis, the accumulation of lysosomal bodies in the upper layers of epidermis has been reported^25^. Added to the complexity, cytosolic calcium possibly activates ER (endoplasmic reticulum) stress that enhances the autophagy in cancer cells through unfolded protein response (UPR)^26^. Recently, ER stress has been shown to induce the lysosome biogenesis and autophagy involving TFEB/TFE3 TFs in an mTORC1-independent manner^27^. Nevertheless, the downstream calcium signaling and the TFs involved in keratinocyte differentiation are largely unknown.

In mammalian cells, UPR is sensed by the three ER resident proteins, namely IRE1 (inositol-requiring enzyme 1), ATF (activating transcription factor) 6, and PERK (protein kinase R like ER kinase). During ER stress, dimerized IRE1 and PERK kinases activate downstream TFs XBP-1 (X-box binding protein 1) and ATF4 respectively. In contrast, ATF6 translocates to the Golgi and then processed to active cytosolic TF after proteolytic cleavage. These TFs crosstalk each other and regulate multiple groups of gene expression including autophagy^28,29^. Moreover, intracellular calcium also modulates UPR pathway to achieve cellular homeostasis^26^. However, the role of ER stress/UPR during calcium-induced keratinocyte differentiation and its regulation on lysosome biogenesis/autophagy is not studied.

To connect keratinocyte differentiation with intracellular calcium, UPR and organelle biogenesis, we used 2 mM CaCl_2_ to differentiate primary neonatal human epidermal keratinocytes (NHEK). Our study illustrated that calcium induces the differentiation and lysosome biogenesis in keratinocytes. Furthermore, intracellular calcium levels are increased during early hours of differentiation that results in activation of ATF6 branch of ER stress. Finally, our study showed that keratinocyte differentiation results in merging and dispersal of fragmented Golgi stacks and colocalization of Golgi tethers with lysosomes. Overall, our study provides a mechanism of keratinocyte differentiation induced by extracellular calcium.

## Results

### Calcium chloride induces keratinocyte differentiation and lysosome biogenesis

**Fig. 1:**
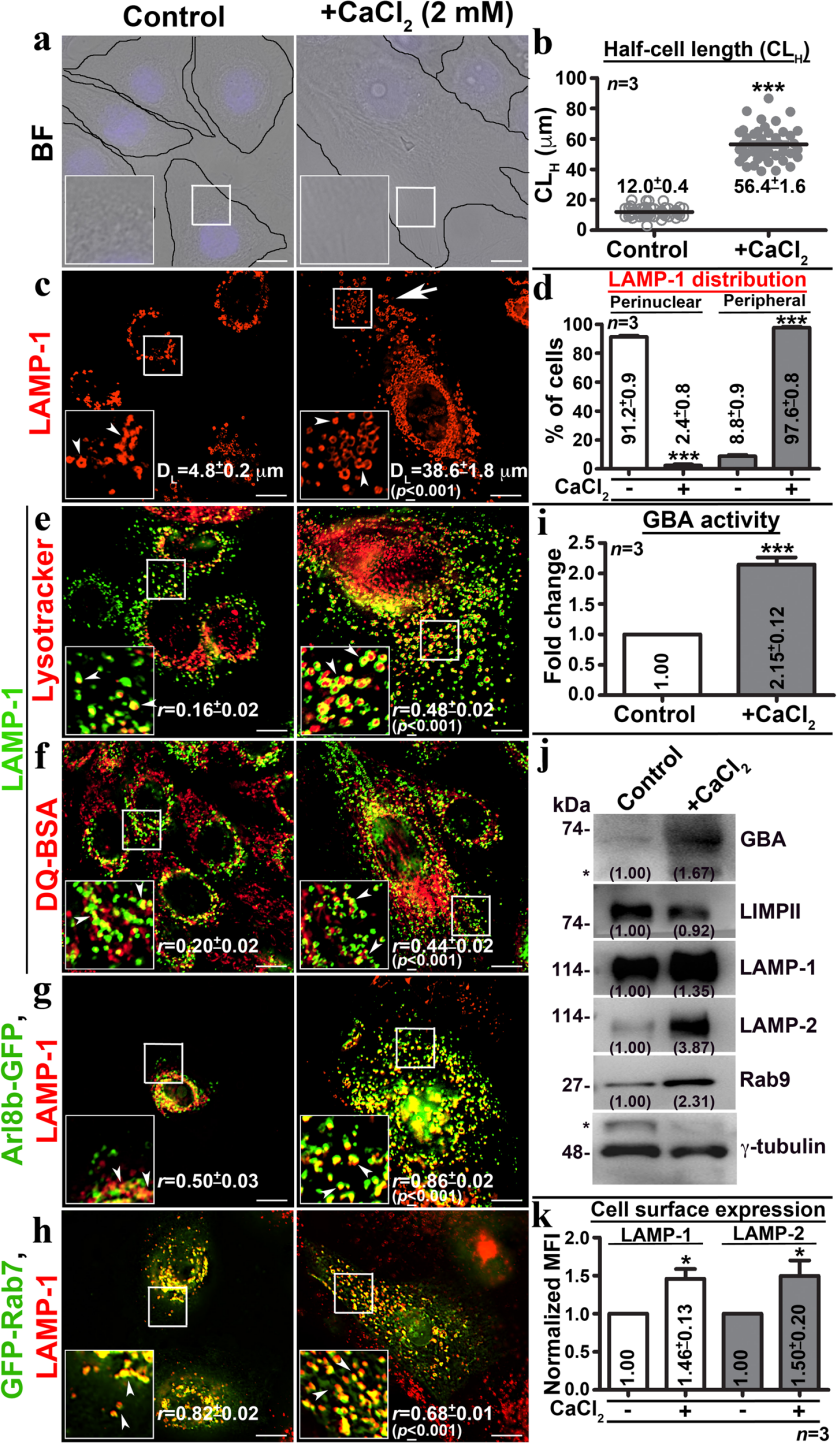
Calcium chloride incubation induces cellular differentiation and increases active enlarged lysosomes in human primary keratinocytes. **a** BF analysis of control and CaCl_2_-incubated (at ~60 h) cells. **b** CL_H_ (in µm) was measured (~60 cells, *n* = 3) in each condition and then plotted (mean ± s.e.m). **c** IFM analysis of LAMP-1 stained control and differentiated keratinocytes. Arrowheads point to LAMP-1 compartments and arrow indicates their distribution and increased number in differentiated cells. D_L_ (in µm) in each condition was measured (~60 cells, *n* = 3) and indicated (mean ± s.e.m.). **d** The cellular distribution of LAMP-1 organelles was quantified visually (~100 cells, *n* = 3) and then plotted (mean ± s.e.m). **e**–**h** IFM analysis of keratinocytes internalized with either lysotracker or DQ-BSA, or transfected with Arl8b-GFP or GFP-Rab7, fixed and stained for LAMP-1. Arrowheads point to the LAMP-1-positive organelles. The degree of colocalization (Pearson’s coefficient, *r*) between markers is indicated separately (mean ± s.e.m., *n* = 3). Nuclei are stained with Hoechst 33258 and the insets are magnified view of the white boxed areas. Scale bars, 10 μm. **i** Glucocerebrosidase (GBA) activity was normalized with cell number and then plotted (fold change in mean ± s.e.m., *n* = 3). **j** Immunoblotting analysis of keratinocytes. The fold change in protein levels is indicated. * Indicates non-specific bands detected by the antibodies. **k** Cell surface levels of LAMP-1 and −2 in control and differentiated cells. Fold change in mean fluorescence intensity (MFI) was calculated (*n* = 3 in quadruplicates) and then plotted (mean ± s.e.m.). *p ≤ 0.05 and ***p ≤ 0.001

Extracellular calcium (1.0–1.8 mM) potentially induces the differentiation of primary human keratinocytes^4,30,31^. However, the mechanisms governing the calcium-induced differentiation process is largely unknown. We incubated NHEK (<6–7 passages) with 2 mM CaCl_2_ (here in +CaCl_2_ or calcium) plated at ~50–60% confluency. Bright-field (BF) microscopy showed increased cell size resembling keratinocyte differentiation post 48 h and the cells appeared as terminally differentiated (tissue with transparent outgrowth on monolayer) after 9 days of CaCl_2_, but not with CaCO_3_ incubation (Supplementary Fig. 1a). Quantification of half-cell length (from nucleus to cell periphery, labeled as CL_H_) showed an increase in cell size by 4.7 folds in 71 ± 7% of calcium-incubated compared to control keratinocytes at ~60 h (Fig. 1a, b and Table 1)^32^. As expected, the nuclear size was increased in CaCl_2_-incubated keratinocytes at ~60 h (Supplementary Fig. 1b). Consistently, the expression of early but not late differentiation markers was significantly increased in CaCl_2_-incubated compared to control keratinocytes (Supplementary Fig. 1c, d)^33–36^. The proliferative cell marker keratin 14 was modestly increased in CaCl_2_-incubated cells (Supplementary Fig. 1d), representing the undifferentiated pool (Table 1). Thus, these studies indicate that 2 mM CaCl_2_ induces early differentiation of primary keratinocytes, resembling the epidermal differentiation^2^.

**Table 1:** Quantification of cellular differentiation and lysosome biogenesis in keratinocytes treated with small molecule inhibitors

S. No.	Treatment		Target	Conc.	Keratinocyte differentiation		
					Cell size (Half-cell length, μm) CL_H_	Lysosome dispersion (μm) D_L_	% of undifferentiated cells (size = <19 μm)
1.	Control				14.2 ± 0.4	4.8 ± 0.2	
2.	CaCl_2_			2 mM	55.1 ± 1.4	38.6 ± 1.8	29.5 ± 7%
3.	**CaCl**_**2**_ **+**	Torin 1	mTORC1	50 nM	67.0 ± 2.2	62.0 ± 2.5	0%
4.		Dorsomorphin	AMPK	5 μM	50.7 ± 1.3	48.6 ± 1.3	3.1%
5.		LY294002	PI3K	50 μM	27.6 ± 1.7	11.1 ± 1.2	28.6%
6.		STO-609-Acetic acid	CaM-KKα/β	1 μg/ml	23.2 ± 1.4	21.3 ± 1.3	38.8%
7.		FK506	Calcineurin	20 μM	25.5 ± 2.4	12.5 ± 2.3	51.0%
8.		Bafilomycin A1	V-ATPase	50 nM	37.6 ± 2.1	18.0 ± 1.9	10.0%
9.		Thapsigargin (Tg)	SERCA	600 nM	22.7 ± 1.8	7.0 ± 0.4	31.3%
10.		4-PBA	ER-Chemical Chaperon	5 mM	19.2 ± 1.0	10.9 ± 1.3	60.4%
11.		4-PBA (L24 h)			34.1 ± 1.0	32.1 ± 1.2	2.0%
12.		4-PBA (F24 h)			18.8 ± 1.3	9.4 ± 0.7	57.1%
13.		4-PBA+Tg			15.6 ± 0.7	5.8 ± 0.4	66.0%
14.		4-PBA+FK506			19.9 ± 1.0	13.8 ± 1.1	41.7%
15.		4-PBA+Torin 1			13.9 ± 0.7	8.9 ± 0.8	82.5%
16.		Torin 1+Tg			19.0 ± 1.0	8.8 ± 0.8	58.0%
17.		Brefeldin A	ARF-1	1 μg/ml	16.1 ± 0.6	6.3 ± 0.3	72.6%

Primary keratinocytes were treated with the indicated compounds (target and their concentrations were listed) along with CaCl_2_ for 48 h, fixed, stained with LAMP-1 and then imaged (see Figs.  3, 5d). In condition 11, cells were treated with CaCl_2_ for 24 h and then incubated with 4-PBA. In condition 12, cells were pre-incubated with 4-PBA for 24 h and then replaced with CaCl_2_. Keratinocyte differentiation and lysosome biogenesis were quantified as half-cell length (CL_H_) and lysosome dispersion (D_L_) respectively, in the indicated treatments (see Figs. 3, 5d; ~60–80 cells, *n* = 3) as described in the materials and methods. Average CL_H_ and D_L_ (in μm) for each treatment were indicated (mean ± s.e.m.). The percentage of undifferentiated cells (having CL_H_ < 19 μm, maximum length observed in proliferative keratinocytes) was calculated from the data presented in Figs. 3b, d and 5e and listed separately in the table

We tested whether the cellular expansion during keratinocyte differentiation alters the biogenesis of intracellular organelles. Immunofluorescence microscopy (IFM) analysis of differentiated keratinocytes showed a dramatic increase in size, number and the peripheral distribution of lysosomes (LAMP-1-positive, see below) compared to early (EEA1-positive) or recycling (STX13-positive) endosomes (Fig. 1c, d and Supplementary Fig. 1e). Quantification of lysosome dispersion (from nucleus to cell periphery, labeled as D_L_) showed 8 folds increase in calcium-added compared to control keratinocytes (Table 1 and Fig. 1c). From here, we used both CL_H_ and D_L_ as a measure of keratinocyte differentiation and lysosome biogenesis, respectively (see below). Further, the LAMP-1-positive organelles in CaCl_2_-treated cells are acidic (lysotracker-positive) and proteolytically active (increased DQ-BSA fluorescence intensity) (Fig. 1e, f for Pearson’s coefficient value, *r*). As expected, these organelles were majorly positive for lysosome-associated proteins (Arl8b-GFP^37^ and Rab9^38^) compared to late endosomal protein (GFP-Rab7^39^) (Fig. 1g, h and Supplementary Fig. 1e), confirming the characteristics of lysosomes. Consistently, the lysosomal enzyme (glucocerebrosidase, GBA) activity was enhanced by 2.15 folds in differentiated compared to control keratinocytes (Fig. 1i). Likewise, the levels of lysosomal proteins (but not the transcripts, see below) (GBA, LAMP-1/−2 or Rab9) but not hydrolase transporters (LIMPII) were significantly increased in differentiated compared to control keratinocytes (Fig. 1j). However, the cell surface expression of LAMP-1 and −2 was moderately increased in CaCl_2_-treated compared to control cells (Fig. 1k). Overall, these studies indicate that the extracellular calcium induces lysosome biogenesis along with keratinocyte differentiation.

### Differentiation linked lysosome biogenesis in keratinocytes is independent of the pathway involving MiT/TFE TFs

**Fig. 2:**
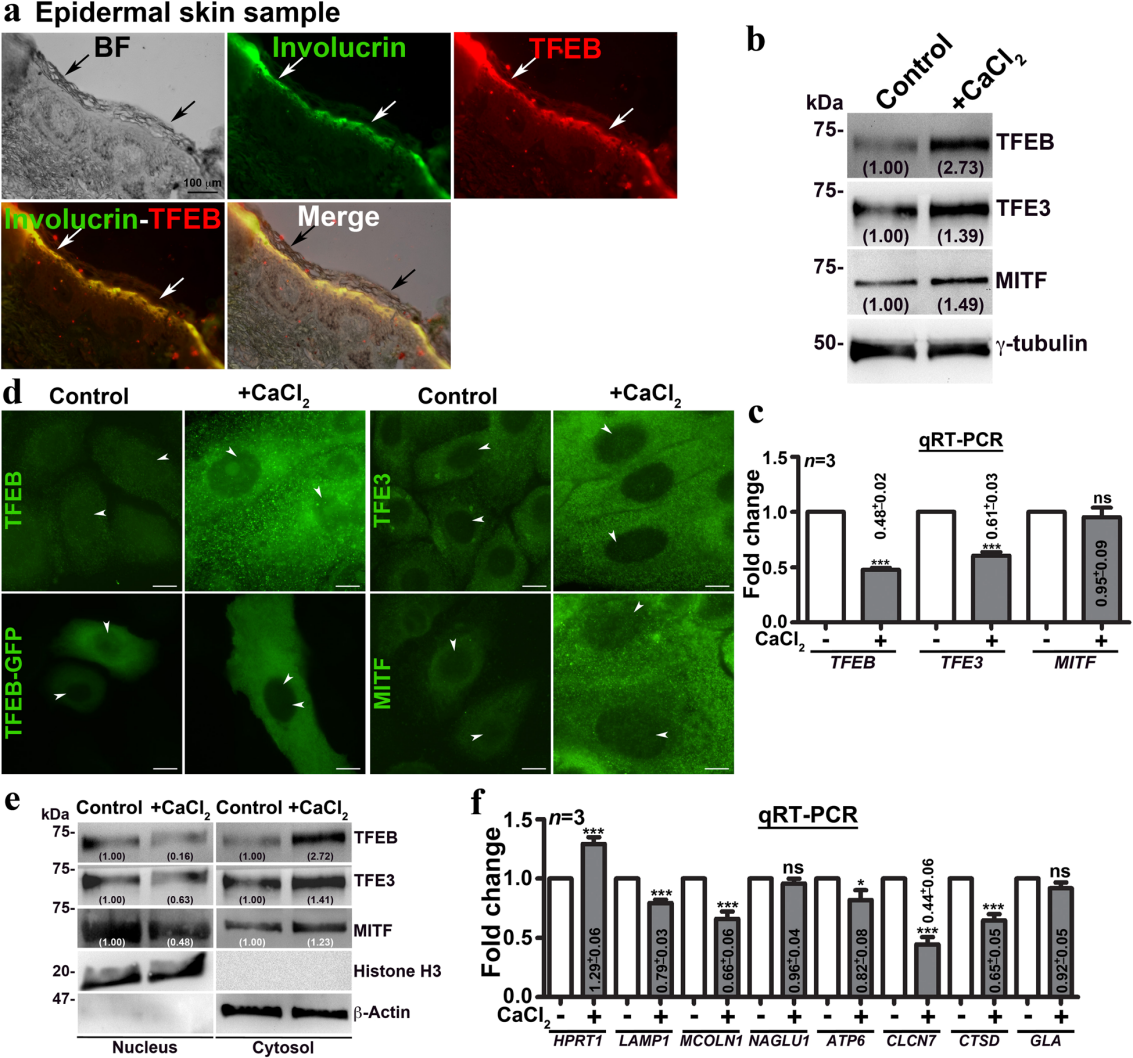
Differentiated keratinocytes of human skin or primary cells increase the expression of MiT/TFE TFs but not their target genes and retained in the cytosol. **a** BF and IFM of epidermal skin sample that was immunostained for involucrin and TFEB. Black arrows point to the cornified skin layer and white arrows indicate the involucrin and TFEB-positive layer. Scale bar, 100 µm. **b**, **c** Immunoblotting and qRT-PCR analyses of MiT/TFE TFs (TFEB, TFE3, and MITF). **d**, **e** IFM and nuclear fractionation analyses of keratinocytes for the localization of TFEB-GFP or endogenous MiT/TFE TFs. Arrowheads point to the localization of TFs to the nucleus. Scale bars, 10 µm. In **b** and **e**, the fold change in protein levels is indicated after normalization with respective loading controls. **f** qRT-PCR analysis of various lysosome biogenesis genes. In **c** and **f**, the fold change (mean ± s.e.m.) in gene expression is indicated (*n* = 3). *p ≤ 0.05; ***p ≤ 0.001 and ns, not significant

Lysosome biogenesis is controlled by MiT/TFE TFs involving TFEB, TFE3, and MITF (microphthalmia-associated TF)^19,21,40^. We investigated the role of these TFs in regulating lysosome biogenesis during keratinocyte differentiation. Histochemistry analysis of skin samples showed enhanced TFEB and LAMP-1 fluorescence intensities in the involucrin-positive (stratum spinosum/granulosum) layer, positioned between the basal and cornified layers of epidermis (Fig. 2a and Supplementary Fig. 1f), indicating an increased lysosome biogenesis in vivo. Similarly, the differentiated keratinocytes displayed enhanced protein levels of MiT/TFE TFs compared to control cells although the transcript levels were reduced or unchanged (Fig. 2b, c). However, IFM and nuclear fractionation analysis showed the localization of GFP-TFEB or the endogenous TFE3 or MITF to nucleus was not increased in calcium-treated compared to control cells (Fig. 2d, e). In line, the expression of several MiT/TFE TF-dependent lysosome biogenesis genes was not altered significantly in differentiated compared to control keratinocytes (Fig. 2f). These results suggest that the enhanced MiT/TFE TFs expression may not contribute to the increased lysosome biogenesis in keratinocytes.

### Keratinocyte differentiation upregulates autophagy and lysosome biogenesis independent of mTOR activity

As reported, MiT/TFE TFs also regulate the expression of autophagy genes^19,41–43^. MTORC1 phosphorylates^21^ and calcineurin dephosphorylates^44^ the MiT/TFE TFs and regulate their cytosol-nuclear localization respectively. Additionally, mTORC1 and 2 are known to modulate the formation of functional epidermis in vivo^17^. Immunoblotting analysis revealed that keratinocytes upregulates mTORC1 activity and the related autophagy components upon calcium addition (Supplementary Fig. 2a). Further, phospho-mTOR (pmTOR) but not mTOR localization to the lysosome membranes^22^ was increased in differentiated compared to control keratinocytes (Supplementary Fig. 2b, c). These results suggest that pmTOR possibly enhance the phosphorylation of MiT/TFE TFs that lead to their retention to the cytosol (Fig. 2d). MTORC2 is known to activate mTORC1 by phosphorylating AKT1^12^. As expected, the phospho-AKT1 level was significantly increased in differentiated keratinocytes (Supplementary Fig. 2a). Chemical inhibition of mTORC1 using Torin 1 enhanced the keratinocyte differentiation to 100% (Table 1) and the cells possess deformed nuclei indicative of initiation of nucleophagy (arrow, Supplementary Fig. 2d). Consistently, nuclear morphology analysis showed increased deformed nuclei in the cells treated with CaCl_2_+Torin 1 compared to CaCl_2_ alone or control (Supplementary Fig. 2e). Surprisingly, TFEB localized majorly to the lysosome membranes upon treatment with Torin 1 in calcium-treated compared to control keratinocytes (Supplementary Fig. 2d). These results indicate that calcium-induced differentiation activates mTORC1 and its inactivation sequesters TFEB onto the lysosome membranes.

Next, we studied the dynamics of autophagosome formation by monitoring the localization and conversion of LC3-I into II. IFM analysis showed increased (by 9 folds) GFP-LC3 puncta (indicative of autophagosomes) in differentiated compared to the control cells (Supplementary Fig. 2f, g). Moreover, colocalization of endogenous LC3 with lysosomes was moderately increased in differentiated compared to control keratinocytes (Supplementary Fig. 2f) in addition to the increased LC3 expression (Supplementary Fig. 2h), indicating an altered autophagy flux in these cells. Conversion of LC3-I to II or the net autophagy flux as measured by using bafilomycin A1 (blocks autophagosome fusion with the lysosome^45^) was dramatically increased in calcium-induced compared to control cells (Supplementary Fig. 2h). Further, the p62 levels^46^ were significantly increased in differentiated keratinocytes (Supplementary Fig. 2a). Altogether, these results suggest that calcium addition to the keratinocytes enhances autophagy flux that may be independent of mTOR activity.

### ER stress regulates keratinocyte differentiation and lysosome biogenesis

**Fig. 3:**
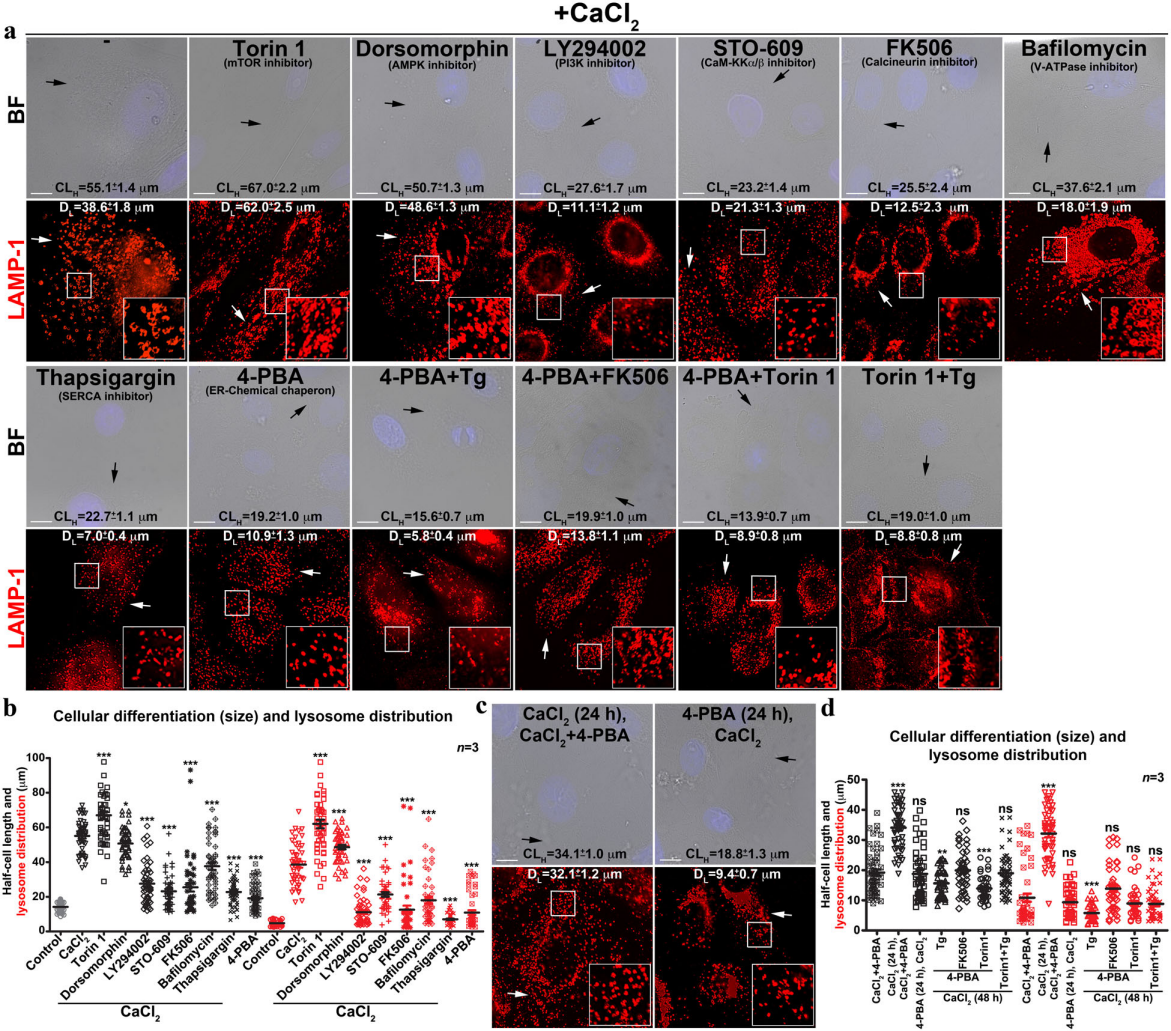
ER stress regulates keratinocyte differentiation and lysosome biogenesis. **a** BF and IFM images of keratinocytes that were co-treated with CaCl_2_ and the indicated compound (at a concentration listed in Table 1) for entire 48 h duration, fixed and stained for LAMP-1. **b** Quantification of CL_H_ and D_L_ in cells shown in (**a**). Statistical analysis between drug treatment and CaCl_2_-alone was indicated. **c** BF and IFM images of keratinocytes those were pre-treated or post-treated with 4-PBA (5 mM) along with CaCl_2_ as described, fixed and then stained for LAMP-1. **d** Quantification of CL_H_ and D_L_ in cells shown in (**c**). In **a** and **c**, black arrows point to the cell limit and white arrows indicate the distribution/morphology of lysosomes. Nuclei are stained with Hoechst 33258 and the insets are magnified view of the white boxed areas. Scale bars, 10 μm. In **b** and **d**, both CL_H_ (black symbols) and D_L_ (red symbols) were quantified as μm from the nucleus towards cell surface (~60–80 cells, *n* = 3 in both) and then plotted. Average CL_H_ and D_L_ (in μm) for each treatment are indicated on IFM images and also in Table 1 (mean ± s.e.m.). *p ≤ 0.05; **p ≤ 0.01; ***p ≤ 0.001 and ns, not significant

To understand the mechanism of calcium-induced keratinocyte differentiation and its molecular link with lysosome biogenesis, we evaluated the role of the key components of calcium signaling and lysosome/Golgi biogenesis by using chemical inhibitors/modulators (Table 1). Interestingly, none of these compounds alone altered the lysosome biogenesis or involucrin fluorescence intensity (Supplementary Fig. 3a, b), indicates that these molecules do not promote the keratinocyte differentiation. To quantify the status of keratinocyte differentiation and lysosome biogenesis, we primarily used the parameters of cell size (CL_H_) and lysosome dispersion (D_L_), respectively. Following, we used quantitative transcript analysis of specific genes (involucrin/keratin 10 for early differentiation, loricrin for late differentiation and LAMP-1 for lysosome biogenesis) to validate these processes. Studies have been shown that cytosolic calcium activates calmodulin and its dependent kinases and phosphatases like calcineurin, which modulates macroautophagy^27,47^. Additionally, PI3K has been shown to play critical role in keratinocyte differentiation^9,16^. Chemical inhibition of calmodulin KKα/β (STO-609-acetic acid) or calcineurin (FK506) activity moderately reduced the calcium-induced keratinocyte differentiation (0.42 folds for calmodulin and 0.46 folds for calcineurin) and lysosome biogenesis (0.55 folds for calmodulin and 0.32 folds for calcineurin) (Fig. 3a, b; Supplementary Fig. 3c and Table 1). In contrast, AMPK (dorsomorphin) inhibition showed no major change in calcium-dependent keratinocyte differentiation/lysosome biogenesis (Fig. 3a, b and Table 1). However, dorsomorphin compound alone induced the expression of early but not late differentiation/LAMP-1 genes in keratinocytes (Supplementary Fig. 3b, c), suggesting a possible pleiotropic effect of dorsomorphin on keratinocyte differentiation. Further, PI3K (LY294002) or V-ATPase (bafilomycin A1) inhibition showed modest reduction in calcium-mediated keratinocyte differentiation (0.5 folds for PI3K and 0.68 folds for V-ATPase) and lysosome biogenesis (0.29 folds for PI3K and 0.47 folds for V-ATPase) (Fig. 3a, b; Supplementary Fig. 3c and Table 1). We noticed bafilomycin treatment enhanced the expression of keratin 10 in keratinocytes (Supplementary Fig. 3c), indicating an indirect effect of this compound on keratin 10 gene expression. As expected, mTORC1 inhibition (Torin 1) showed accelerated differentiation/lysosome biogenesis compared to CaCl_2_-incubated cells (Fig. 3a, b and Table 1). In line, the expression of both early (except keratin 10) and late differentiation genes was significantly increased with Torin 1 alone and further enhanced in presence of CaCl_2_ (Supplementary Fig. 3b, c), suggesting an additive effect of Torin 1 on calcium-induced keratinocyte differentiation.

Co-treatment of keratinocytes with ER stress modulator thapsigargin (Tg, an inhibitor of SERCA/sarco-ER calcium ATPase-calcium pump^48^) and CaCl_2_ significantly reduced the differentiation (0.41 folds) and completely abolished the lysosome biogenesis (0.18 folds) compared to CaCl_2_ alone (Fig. 3a, b and Table 1; see below). Surprisingly, ER stress attenuator 4-PBA (4-Phenylbutyric acid, a chemical chaperon that attenuates ER stress by increasing protein folding)^49^ did not enhance either the calcium-dependent keratinocyte differentiation (0.35 folds) nor lysosome biogenesis (0.28 folds) compared to CaCl_2_ alone (Fig. 3a, b and Table 1). These results are consistent with the reduced expression of keratinocyte differentiation genes in both the conditions (Supplementary Fig. 3c). To understand the role of ER stress in keratinocyte differentiation and its concomitant lysosome biogenesis, we have incubated the cells with only CaCl_2_ for 24 h and then co-incubated with 4-PBA for another 24 h or pre-treated the cells only with 4-PBA for 24 h and then incubated with CaCl_2_ (Fig. 3c, d and Table 1). Pre-treatment of cells with 4-PBA inhibited both keratinocyte differentiation and lysosome biogenesis equally to that of 4-PBA+CaCl_2_ treated cells (Table 1). In contrast, post treatment of 4-PBA did not show any strong effect on CaCl_2_-induced keratinocyte differentiation/lysosome biogenesis (Fig. 3c, d and Table 1). Based on these results, we hypothesize that extracellular calcium or initial cellular calcium signaling very likely generates ER stress that possibly drives the differentiation of primary human keratinocytes. 4-PBA has been shown to attenuate the Tg-induced ER stress in tumor microenvironment^50^. Co-treatment of keratinocytes with Tg and 4-PBA with CaCl_2_ showed significant reduction in cellular size (differentiation) as well as lysosome biogenesis compared to Tg+CaCl_2_ or 4-PBA+CaCl_2_-incubated cells (Fig. 3a, d and Table 1). These results suggest that 4-PBA-mediated ER stress attenuation is not sufficient to rescue the Tg-mediated blockade of cellular differentiation. Additionally, coincubation of keratinocytes with 4-PBA, FK506 and CaCl_2_ showed similar level of differentiation/lysosome biogenesis as observed in cells treated with 4-PBA+CaCl_2_ or FK506+CaCl_2_ (Fig. 3a, d and Table 1). Moreover, Torin 1-mediated enhanced keratinocyte differentiation (including lysosome biogenesis) was drastically reduced upon coincubation with 4-PBA or Tg along with CaCl_2_ (Fig. 3a, d and Table 1). These results indicate that attenuation of ER stress at early hours by using 4-PBA or an additional ER stress induced by Tg may block the keratinocyte differentiation. Additionally, we speculate that calcium refilling through SERCA pump is required for both keratinocyte differentiation and lysosome biogenesis.

### Cytosolic calcium activates UPR TFs and modulates lysosome biogenesis during keratinocyte differentiation

**Fig. 4:**
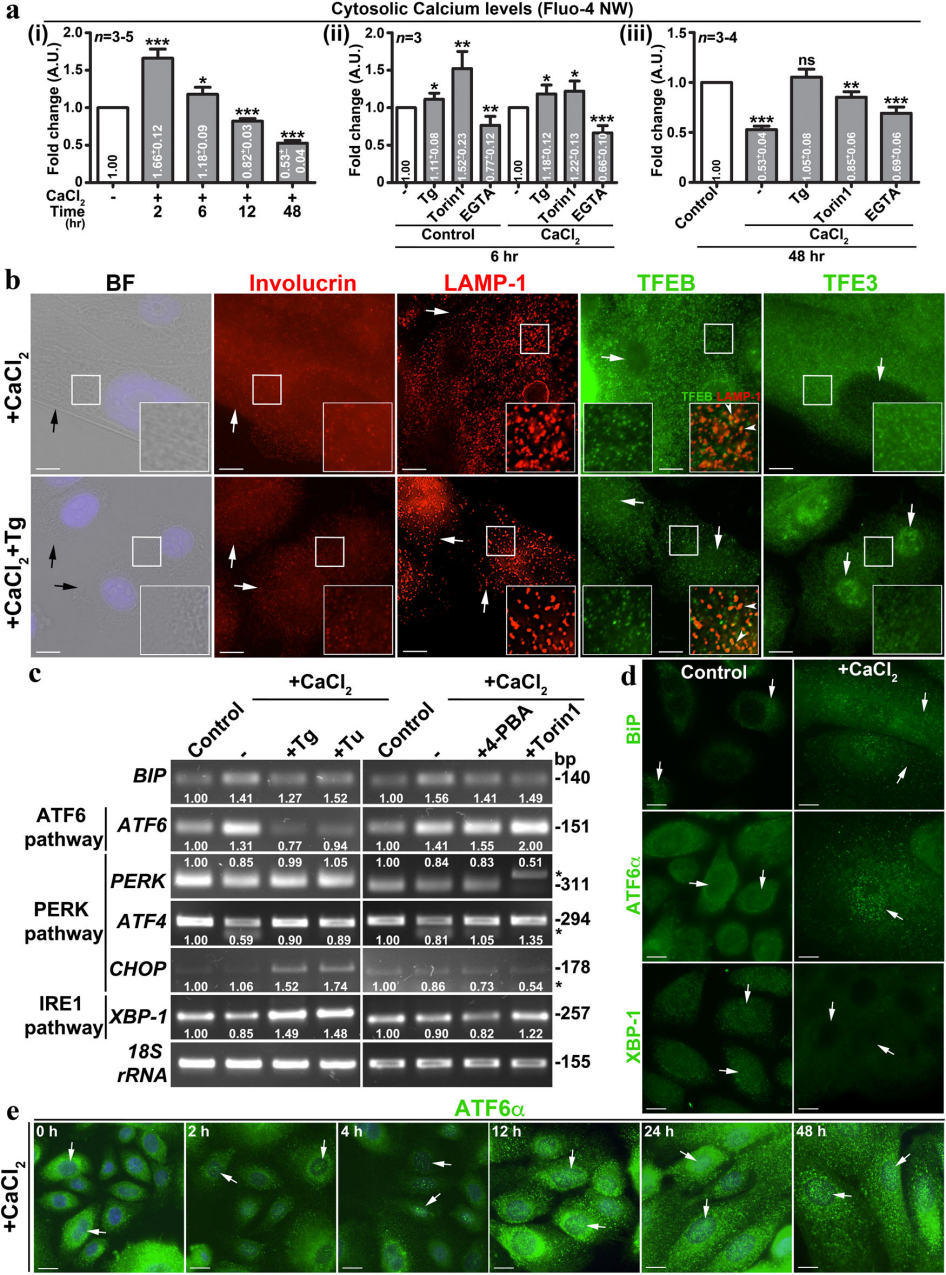
Differentiation of keratinocytes elevates cytosolic calcium at early phase and activates UPR TF ATF6α. **a** Measurement of intracellular calcium in keratinocytes at different time points of differentiation. In (i), cells were treated with CaCl_2_ for respective time points. In (ii and iii), cells were incubated with the indicated compounds alone or coincubated with CaCl_2_ for the respective time points. In all conditions, cells were incubated with Fluo-4 NW for 2–3 h either prior to or during the treatment condition. The fold change (mean ± s.e.m.) in fluorescence intensities is indicated. Note that EGTA was used as the negative control in the experiments. *p ≤ 0.05; **p ≤ 0.01; ***p ≤ 0.001 and ns, not significant. **b** BF and IFM analysis of keratinocytes that were incubated with CaCl_2_ alone or CaCl_2_ with Tg for 48 h. Black arrows point to the cell limit. White arrows indicate the expression of proteins or distribution of lysosomes or localization of respective TFs to the nucleus. Arrowheads point to the localization of TFEB to the lysosomes. **c** Transcript analysis of ER stress genes in the keratinocytes that were treated with either CaCl_2_ alone or CaCl_2_ with the indicated compounds for 48 h. The fold change in gene expression is indicated. * Indicates non-specific bands. **d**, **e** IFM analysis of keratinocytes that were incubated with CaCl_2_ for 48 h (**d**) or indicated time points (**e**). White arrows indicate the expression of BIP or localization of respective TFs to the nucleus. Nuclei are stained with Hoechst 33258 and the insets are magnified view of the white boxed areas. Scale bars, 10 μm

We evaluated the role of cytosolic calcium in the process of keratinocyte differentiation. ER calcium pumps maintain the intracellular calcium concentration/gradient either by releasing calcium to the cytosol (by IP3R, RYR/ryanodine receptor) or by pumping extra calcium into ER (by SERCA). Interestingly, the cytosolic calcium levels were significantly higher in the initial two hours of keratinocyte differentiation and restored to or reduced than the basal calcium levels at 48 h (Fig. 4ai), suggesting a role for early calcium flux that may lead to the ER stress. Moreover, this data suggest that differentiated keratinocytes maintain lower calcium concentration than control cells (Fig. 4ai), in spite their constant exposure to extra cellular high calcium. Upon treatment with Tg for 6 h, the peak calcium flux observed in differentiated keratinocytes was abolished and appeared as equivalent to that of the control cells (Fig. 4aii). Consistently, calcium chelator EGTA had no effect (at 6 h) on intracellular calcium flux in the keratinocytes with or without addition of extracellular calcium (Fig. 4aii). Calcium release from the ER has been shown to modulate the mTOR activity and TFEB/TFE3-mediated lysosomal gene expression^27^. Interestingly, treatment of keratinocytes with mTOR inhibitor Torin 1 (for 6 h) moderately increased the cytosolic calcium both in Torin 1-alone and CaCl_2_+Torin 1-incubated cells compared to only CaCl_2_-treated cells (Fig. 4ai, ii). As expected, intracellular calcium level was lower than the basal level (slightly higher than the CaCl_2_-alone) upon treatment of keratinocytes with CaCl_2_+Torin 1 for 48 h (Fig. 4aiii). Thus, these results explain the enhanced keratinocyte differentiation observed with CaCl_2_+Torin 1 compared to only CaCl_2_-incubated cells (Table 1). Overall, these studies suggest that initial calcium peak at 2 h possibly required for keratinocyte differentiation in addition to the function of SERCA pump. Furthermore, we hypothesize that SERCA pump possibly maintains lower cytosolic calcium concentration during keratinocyte differentiation.

We tested whether the inhibition of SERCA function alters differentiation of keratinocytes. Tg-treated calcium-incubated keratinocytes showed reduced involucrin expression and cell size compared to differentiated cells (Fig. 4b and Table 1). Consistently, LAMP-1-positive lysosomes were reduced in CaCl_2_+Tg-treated cells (Fig. 4b). As expected, the localization of TFEB to the nucleus was not altered (arrows), but its localization to the lysosomes was moderately reduced (arrowheads) in CaCl_2_+Tg-treated keratinocytes (Fig. 4b, inset of TFEB panel). In contrast, the nuclear localization of TFE3 was enhanced upon treatment of keratinocytes with Tg and CaCl_2_ (Fig. 4b), however, it did not contribute to the increased lysosome biogenesis or keratinocyte differentiation (Table 1 and Fig. 2f). Increased cytosolic calcium has been shown to upregulate the UPR^27^. We evaluated whether ER stress is enhanced during keratinocyte differentiation process. Transcript analysis showed moderate increase in BIP expression in differentiated kerationcytes (Fig. 4c and Supplementary Fig. 4a). Interestingly, increased BIP transcript levels in the differentiated cells were not further enhanced with ER stress inducers Tg or Tu (tunicamycin), nor reduced with ER stress attenuator 4-PBA or mTOR inhibitor Torin 1 (Fig. 4c and Supplementary Fig. 4a). Consistently, differentiated keratinocytes showed a notable increase in BIP and calnexin (ER chaperon) protein levels (Fig. 4d and Supplementary Fig. 4b, 5a), suggesting a moderate upregulation of ER stress during keratinocyte differentiation.

Next, we examined the status of three branches of UPR during calcium-induced keratinocyte differentiation. Transcript analysis showed increased expression of ATF6 but not PERK (PERK, ATF4, CHOP)- or IRE1 (XBP-1)-dependent pathway genes in differentiated keratinocytes (Fig. 4c and Supplementary Fig. 4a). Interestingly, the upregulation of ATF6 transcripts upon calcium-treatment was similar even after the incubation of cells with 4-PBA or Torin 1 (Fig. 4c and Supplementary Fig. 4a). However, the treatment of calcium-incubated cells with 4-PBA or Torin 1 did not alter the transcription profile of PERK or IRE1-pathway genes (Fig. 4c and Supplementary Fig. 4a). In contrast, Tg or Tu treatment during keratinocyte differentiation significantly reduced the transcript levels of ATF6 (Fig. 4c and Supplementary Fig. 4a), consistent with a block in keratinocyte differentiation (Fig. 3a, b and Supplementary Fig. 3c, Table 1, data not shown for tunicamycin). Further, moderately enhanced CHOP (PARK-pathway) and XBP-1 (IRE1 pathway), but not PERK or ATF4 transcripts were observed upon treatment with Tg or Tu during differentiation (Fig. 4c and Supplementary Fig. 4a). Active ATF6 TF^51^ has been shown to regulate the lysosome biogenesis and autophagy^52^. Immunoblotting analysis showed marginally increased ATF6 TF and PERK protein levels compared to IRE1α or spliced XBP-1 in differentiated keratinocyte cells (Supplementary Fig. 4b). Interestingly, the nuclear localization of ATF6 but not XBP-1 was increased in differentiated keratinocytes compared to control cells (Fig. 4d). As expected, time kinetics studies showed enhanced localization of ATF6 to the nucleus between 12 and 48 h of calcium-incubated keratinocytes (Fig. 4e). Thus, these studies suggest that ATF6 TF possibly play a key role in calcium-dependent keratinocyte differentiation.

### ER stress-dependent lysosomes possibly derived from Golgi during keratinocyte differentiation

**Fig. 5:**
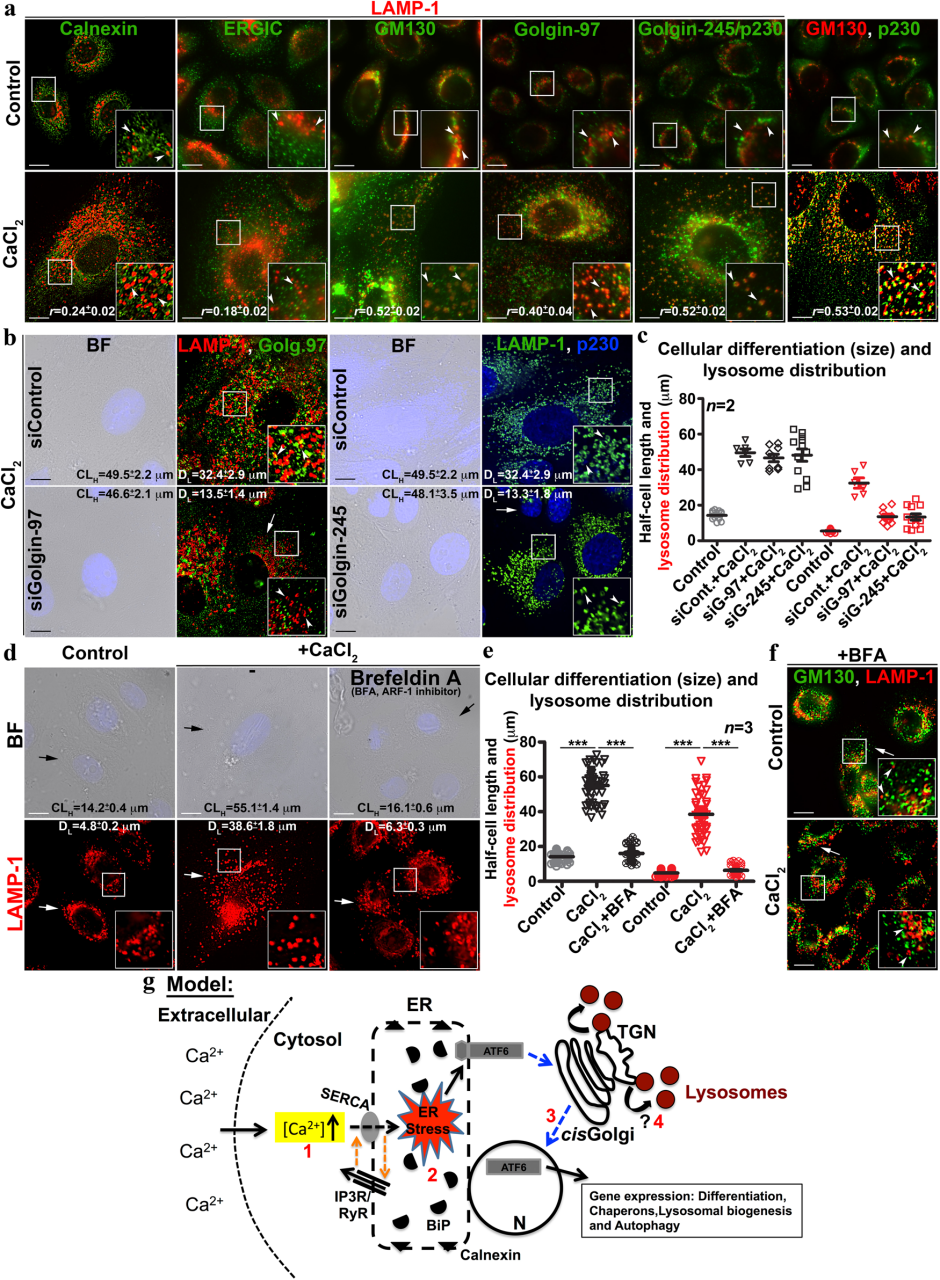
Keratinocyte differentiation causes fragmentation and dispersal of Golgi and increases colocalization of Golgi tethering proteins with lysosomes. The model illustrating the mechanism of keratinocyte differentiation and lysosome biogenesis. **a** IFM analysis of control and differentiated keratinocytes for the localization of ER, transitional ER and Golgi-associated proteins with respect to the lysosomes or the organization of Golgi apparatus (indicated by arrowheads). The degree of colocalization (Pearson’s coefficient, *r*) between the markers was indicated separately (mean ± s.e.m., *n* = 3). **b** BF and IFM images of keratinocytes those were transfected with respective siRNA. White arrows indicate the loss in fluorescence intensity of Golgi-tethering proteins. Arrowheads point to the localization of LAMP-1 with respect to the Golgi-associated proteins. **c** Quantification of CL_H_ and D_L_ in cells shown in (**b**). **d** BF and IFM images of keratinocytes that were treated with brefeldin A (1 μg/ml) alone or with CaCl_2_ for entire 48 h duration. Black arrows point to the cell limit and white arrows indicate the distribution/morphology of lysosomes. **e** Quantification of CL_H_ and D_L_ in cells shown in (**d**). In **c** and **e**, both CL_H_ (black symbols) and D_L_ (red symbols) were quantified as μm from the nucleus towards cell surface (~20 cells, *n* = 2 in **c** and ~60–80 cells, *n* = 3 in **e**) and then plotted. Average CL_H_ and D_L_ (in μm) for each treatment were indicated (mean ± s.e.m.) on IFM images. ***p ≤ 0.001. **f** IFM analysis of keratinocytes those were described in (**d**). White arrows indicate the loss in dispersal of Golgi and loss in colocalization between LAMP-1 and GM130. Arrowheads point to the localization of LAMP-1 with respect to GM130. Nuclei are stained with Hoechst 33258 and the insets are magnified view of the white boxed areas. Scale bars, 10 μm. **g** Model: Prolonged exposure of keratinocytes to CaCl_2_ increases intracellular calcium [Ca^2+^] levels (1) within 2 h and possibly elevates ER stress (2) due to altered ER calcium refilling cycle/gradient (orange arrows). Enhanced ER stress promote the Golgi trafficking and processing of ATF6α in to an active UPR TF (3), which possibly initiates the keratinocyte differentiation program. During this process, Golgi compartments merged and generate the enlarged globular lysosomes (4), which probably senses intracellular signaling and balances calcium concentration by acting as reservoir. ? indicates the process requires future investigation

To investigate the origin of ER stress-dependent lysosomes, we evaluated the role of other organelles in the differentiated keratinocytes. IFM analysis showed cellular distribution of early, recycling and late endosomes was not affected in differentiated keratinocytes (Supplementary Fig. 1g). Interestingly, the dispersal and fragmentation of both *cis-* and *trans-*Golgi (labeled with Golgi-tethering proteins GM130 and Golgin-97 or Golgin-245/p230, respectively), but not transitional ER (marked by ERGIC-53) were dramatically increased in differentiated keratinocytes compared to control cells (Fig. 5a and Supplementary Fig. 5b–f). Moreover, keratinocyte differentiation showed increased colocalization of GM130 with Golgin-245 suggesting a merge between the Golgi compartments (Fig. 5a and Supplementary Fig. 5f). To our surprize, cohort of fragmented *cis*- and *trans*-Golgi (in the peripheral cytosol), but not the ER or transitional ER showed colocalization with LAMP-1-positive lysosomes in differentiated cells compared to control cells (Fig. 5a and Supplementary Fig. 5a–e), indicating that either these lysosomes are originated from Golgi compartments or Golgi-vesicles accumulate the lysosomal cargo/hydrolases. Cargo internalization experiments showed the trafficking of degradative soluble cargo such as fluorescein-dextran or DQ-BSA to lysosomes was unaffected in differentiated keratinocytes (Fig. 1f and Supplementary Fig. 5g). Interestingly, the internalized dextran was trafficked to Golgin-positive lysosomes at cell periphery but not to the perinuclear Golgi (Supplementary Fig. 5g). These results indicate that the classical endocytic route to the lysosomes or the bonafide nature of lysosomes are not altered in differentiated keratinocytes. SiRNA-mediated depletion of either Golgin-97 or Golgin-245/p230 (causes dispersion of Golgi and affects Golgi positioning indirectly^53,54^) did not inhibit the calcium-induced differentiation nor the lysosome biogenesis in keratinocytes. However, lysosomes were clustered at perinuclear region and appeared in slightly smaller size in both golgin-97/245-knockdown cells compared to control cells (Fig. 5b, c and Supplementary Fig. 5h, i), suggesting that Golgi tethers do not play a key role in keratinocyte differentiation/lysosome biogenesis. Surprisingly, the inhibition of Golgi function using brefeldin A (BFA, inhibits ARF-1 activity) completely abolished the cellular differentiation and lysosome biogenesis in keratinocytes (Fig. 5d, e and Table 1). Moreover, the dispersed Golgi upon treatment of keratinocytes with BFA showed no colocalization with lysosomes either in control or CaCl_2_-treated cells (Fig. 5f and Supplementary Fig. 5j), indicating that Golgi dispersal is not sufficient to accumulate lysosomal cargo such as LAMP1 in those vesicles. Overall, these studies revealed that intact Golgi or its associated secretion possibly regulate the biogenesis of ER stress-dependent lysosomes during keratinocyte differentiation. In a nutshell, these studies illustrated a mechanism by which ATF6-arm of UPR control both the keratinocyte differentiation and lysosome biogenesis, which are essential for the epidermal homeostasis.

## Discussion

Cellular differentiation is one of the adaptive survival mechanisms in response to extracellular stimuli. Epidermal keratinocytes of upper basal layer encounter both nutritional and ionic (calcium) stress that lead to a change in cell fate from proliferation to differentiation^2,55^ in addition to an increase in intracellular digestion through autophagy^7,8^. Extracellular high calcium has been shown to play a key role in inducing keratinocyte differentiation^2^, but its role in upregulating cellular clearance activity is poorly understood. Our in vitro model of keratinocyte differentiation using CaCl_2_ illustrated a dramatic increase in the number and size of functional lysosomes, which possibly maintains cellular digestion/macroautophagy. Moreover, our study demonstrated that an initial rise (early as 2 h) in cytosolic calcium possibly triggers ATF6-arm of UPR that enhances lysosome biogenesis independently of mTOR and MiT/TFE TFs. Consistently, inhibition of ER/Golgi function, but not the lysosome/-associated signaling activity abolished the lysosome biogenesis and keratinocyte differentiation. Thus, our study provides molecular mechanism of keratinocyte differentiation in response to extracellular high calcium, possibly required to maintain the epidermal homeostasis.

How extracellular high calcium lead to lysosome biogenesis? We predict that keratinocytes balance the calcium stress by maintaining the active calcium gradient between extracellular environment—cytosol—intraorganelles such as ER, lysosomes etc.^56^. Elevated calcium levels in the cytosol induces multiple calcium-dependent signaling pathways that may lead to the activation of apoptosis^57,58^. To escape the programmed cell death, cells are very likely to accommodate excess intracellular calcium in membrane bound organelles such as lysosomes in addition to ER and mitochondria. Several observations in our study support this hypothesis: (1) the cytosolic calcium level was lower in differentiated compared to proliferating keratinocytes; (2) inhibition of SERCA pump using Tg significantly reduced the lysosome biogenesis and differentiation; (3) chemical inhibition of calmodulin or calcineurin showed moderate effect on differentiation and (4) calcium-dependent activation of ATF6 enhances organelle biogenesis such as lysosomes and autophagosomes. In line with these results, a co-ordinated role between cell surface calcium pumps with ER SERCA in maintaining the low cytosolic calcium concentration has been implicated previously^59^. Thus, lysosome biogenesis during keratinocyte differentiation is one of the possible mechanisms to nullify the nutritional and ionic stresses in the epidermis.

Differentiated keratinocytes in the upper layers of epidermis gradually lose intracellular organelles by increasing macroautophagy^7,8^. In turn, the enhanced autophagy requires functional lysosomes to maintain its turnover. But it is unclear how these lysosomes are produced during keratinocyte differentiation. Our study provides a model (Fig. 5g) in which initial intracellular calcium rise (within 2 h) creates ER stress (step 1 and 2), which then activates ATF6-arm of UPR and generates active ATF6 TF (step 3). ATF6 localize to the nucleus and possibly upregulate the genes for differentiation, lysosome biogenesis, autophagy etc. In parallel, cells possibly promote the generation and dispersal of functional lysosomes from Golgi (step 4) to reduce the extracellular calcium-induced ER stress. Thus, the process of lysosome biogenesis during keratinocyte differentiation follow a non-canonical secretory pathway involving ER stress rather than mTOR-MiT/TFE TFs-dependent classical biogenesis process^20–22^. Several results of our study support this pathway: (a) increased ATF6α TF levels and its localization to the nucleus over time; (b) retention of MiT/TFE TFs to the cytosol due to enhanced mTOR activity; (c) localization of pmTOR to the lysosomes; and (d) colocalization of Golgi tethers with bonafied lysosomes. Additionally, our model of lysosome biogenesis support the balance required for the turnover of enhanced autophagy flux observed during keratinocyte differentiation.

Is lysosome biogenesis essential for keratinocyte differentiation or it is a cause and effect of the differentiation program? Several studies have reported that autophagy is the key process known to affect the epidermal differentiation and skin architecture^7,8,24,60^. To our surprise, master regulatory TFs of autophagy and lysosome biogenesis showed negligible effect on keratinocyte differentiation. This led us identifying a non-canonical pathway involving ER stress that regulates lysosome biogenesis and keratinocyte differentiation. Chemical inhibition of pathways revealed that modulation of ER stress or Golgi function by using Tg/4-PBA or brefeldin A respectively, completely abolished the keratinocyte differentiation and lysosome biogenesis (Fig. 3 and Table 1), however, alteration of lysosome acidity using bafilomycin A1 had no effect on these processes (only D_L_ is reduced, see Fig. 3 and Table 1). Thus, our studies provide evidence that ER stress is essential for the lysosome biogenesis to compensate the increased autophagic flux during keratinocyte differentiation. Overall, these studies correlate to the calcium stress observed in the upper basal layer of epidermis, possibly promotes keratinocyte differentiation as an adaptive mechanism, which further compensate the nutrient stress noticed in these layers. Moreover, these studies may help in understanding the defective epidermal homeostasis or architecture observed in skin diseases such as psoriasis and atopic dermatitis.

## Materials and methods

### Reagents and antibodies

All chemicals and reagents were purchased either from Sigma-Aldrich (Merck) or ThermoFisher Scientific (Invitrogen). Torin 1 was purchased from Tocris Bioscience. Tissue culture reagents such as EpiLife medium, HKGS (Human Keratinocyte Growth Supplement), Trypsin neutralizer solution and reagents including LysoTracker Red DND-99, DQ-BSA, Fluo-4 NW, Fluorescein-conjugated-Dextran were obtained from ThermoFisher Scientific (Invitrogen). The following commercial polyclonal and monoclonal antisera were used (m, mouse; h, human and r, rat proteins). Anti-mLIMPII (ab16522) was from Abcam; anti-rGM130 (610822) and anti-hp230/Golgin-245 (611281) were from BD Biosciences; anti-pAKT (9271), anti-Beclin-1 (3495); anti-BiP (3177), anti-Calnexin (2679), anti-CHOP (2895), anti-4E-BP1 (9452), anti-EEA1 (3288), anti-IRE1α (3294), anti-PERK (5683), anti-Rab9 (5118), anti-Raptor (2280); anti-Rictor (2114); anti-LC3A/B (4108), anti-pS6K (T389; 9234), anti-SQSTM1 (p62; 5114), anti-TFEB (4240), anti-mTOR (2983) and anti-p-mTOR (S2448; 5536) were from Cell Signalling Technology; anti-hLAMP-1 (H4A3) and anti-hLAMP-2 (H4B4) were from Developmental Studies Hybridoma Bank; anti-hATF-6α (sc-22799), anti-hInvolucrin (sc-21748), anti-hMITF (sc-10999) and anti-mXBP-1 (sc-7160) were from Santa Cruz Biotechnology; anti-β-actin (A5441), anti-hGBA (G4171), anti-HA (H3663), anti-Histone H3 (H9289), anti-TFE3 (HPA023881) and γ-tubulin (GTU88; T6557) from Sigma-Aldrich. Antisera to Golgin97, ERGIC (Michael S. Marks, University of Pennsylvania, Philadelphia, USA) and STX13 (Andrew Peden, University of Sheffield, Sheffield, UK) were obtained as gift from respective laboratories. All secondary antibodies were either from Invitrogen or Jackson Immunoresearch.

### Plasmids

Arl8b-GFP: Human Arl8b was PCR amplified from cDNA derived from HeLa cells, digested and subcloned into XhoI and BamHI sites of pEGFP-N1 vector (Clontech). TFEB-GFP: Human TFEB was PCR amplified from HeLa cells cDNA, digested and subcloned into SalI and BamHI sites of pEGFP-N3 vector (Clontech). GFP-Rab7 was a kind gift from M. Sharma, IISER Mohali, India and GFP-rLC3 (21073) was obtained from Addgene.

### Cell culture, differentiation, transfection, and drug treatment

Neonatal human epidermal keratinocytes (NHEK, Indian origin) were purchased from Lonza or Invitrogen. Early passaged cells (<7 from initial plating) were maintained in EpiLife serum free medium (contains 60 µM CaCl_2_) supplemented with HKGS. Cells were plated on 0.002% collagen coated surface for better adherence and then incubated at 37 °C with 10% CO_2_. For differentiation, keratinocytes at a confluency of 50–60% were supplemented with 2 mM CaCl_2_ for 2–9 days and changed the medium every 24 h. We observed more than 70% (see Table 1) cells showed differentiation characteristics starting from 48 h. Cells between 48 and 72 h of differentiation were used for all experiments. DNA vectors were transfected into the cells by using Lipofectamine 2000 (Invitrogen) according to the manufacturer’s protocol. Cells were fixed, immunostained and imaged as described previously^61^. For drug treatment, cells were incubated with respective drug concentrations (listed in Table 1, obtained from the literature) with or without 2 mM CaCl_2_ for 48 h, fixed and then stained. Note that cells were replenished with their respective medium at 24 h. Moreover, we used lower drug concentrations to avoid reversible effect of the compound within 48 h. To equivalize the time, cells were treated with drugs throughout the 48 h time period. Half-cell length (CL_H_) as parameter to measure differentiation and dispersion of lysosomes (D_L_) as parameter to measure the lysosome biogenesis were quantified (see below) in µm (from the nucleus to cell periphery) and then compared with CaCl_2_-treated cells. For lysotracker staining, cells on glass coverslips were incubated with 50–75 nM of lysotracker in growth medium for 30 min at 37 °C in an incubator maintained at 10% CO_2_. Similarly for DQ-BSA internalization, cells were incubated with 10 µg/ml of DQ-BSA in a complete medium for 2 h, washed once with 1 × PBS and then incubated in plain medium for 4 h. Likewise, cells were incubated with 0.5 mg/ml of fluorescein-conjugated-dextran in a complete medium for 6 h, washed twice with 1 × PBS and then chased for 12 h in plain medium. Finally, cells were fixed with 3% paraformaldehyde solution, stained and imaged. For siRNA transfection, cells on coverslips were transfected with 120 μM of respective siRNA (Supplementary Table 1) per well in a 12 well plate using Oligofectamine (Invitrogen) according to the manufacturer’s protocol. Post 48 h of transfection, cells were fixed, stained, and imaged.

### Epidermal skin samples and immunohistochemistry

Epidermal foreskin samples were obtained as waste discard post cosmetic surgery with informed patient consent and local ethical committee approval. These samples were embedded in paraffin blocks, sectioned into 5 μm thick slices using microtome (Leica RM 2155) and then placed on poly-L-lysine coated slides (Sigma-Aldrich). Sections were deparaffinized using xylene followed by rehydration sequentially with ethanol (100%, 90%, 70%) and then with distilled water. Slices were further treated with 10 mM sodium citrate buffer pH 6.1 and then incubated in 1 × PBS for 45 min followed by an incubation in 1.5% blocking serum in PBS (Santa Cruz Biotechnology, sc-2043) for 1 h at room temperature under moist condition. After the removal of excess blocking serum, the sections were immunostained with indicated antibodies (1:20 dilution in blocking serum) for overnight at 4 °C. Slides were rinsed with 1 × PBS containing 0.05% Tween 20 for three times (5 min each) and then stained with respective secondary antibodies for 1 h at room temperature. Finally, slides were washed and then imaged.

### Lysosomal enzyme (glucocerebrosidase, GBA) activity assay

Keratinocytes were seeded at 70–80% confluence in black 96 well flat-bottom black plate (Corning) and performed the Resazurin cell viability assay following intact cell lysosomal β-glucosidase assay as described previously^62^. Briefly, 10 μl of Resazurin (Sigma-Aldrich, 10 mg/ml made in 1 × PBS) in 90 µl of medium was added to the cells and then incubated at 37 °C for 6–7 h. The fluorescence intensity was measured at 530 nm excitation and 590 nm emission using Tecan multi-mode plate reader (Infinite F200 Pro). Next, the same cells were washed twice with 1 × PBS and then incubated with 50 µl of 3 mM MUD (4-methyl umbelliferyl-β-D-glucopyranoside, made in 0.2 M sodium acetate buffer pH 4.0) at 37 °C for 3 h. Further, the assay was stopped by adding 150 µl of 0.2 M glycine buffer pH 10.8 and then measured the fluorescence intensity of liberated 4-methylumbelliferone (excitation at 365 nm and emission at 445 nm) using Tecan multi-mode plate reader. Finally, the lysosomal enzyme activity per well was normalized with respective cell viability and then plotted.

### Measurement of intracellular calcium levels

Primary keratinocytes were seeded at 70–80% confluence in black 96 well flat bottom plate (Corning) and incubated with CaCl_2_ for different time intervals or treated with Tg or Torin 1 or EGTA (as control) in combination with CaCl_2_ for 6 h or 48 h. Intracellular free calcium was measured using Fluo-4 NW calcium assay kit (Molecular probes, F36206). Briefly, Fluo-4 NW dye was added to the cells 2–6 h prior to the end of time point. The total cellular fluorescence was measured at 516 nm with an excitation of 494 nm using Tecan multi-mode plate reader. The emission fluorescence intensity values were normalized with the cell numbers measured by Resazurin (Sigma-Aldrich)-based cell viability assay. The fold change in fluorescence intensity between the treatment and control was measured and then plotted.

### Cell surface expression using flow cytometry

Cell surface expression of LAMP-1 and LAMP-2 was measured as described previously^63^. Briefly, cells were harvested, washed once with 1 × PBS, suspended in ice-cold growth medium (supplemented with 25 mM HEPES pH 7.4) containing anti-LAMP-1, anti-LAMP-2 or anti-Tac (7G7.B6 from ATCC, as negative control) and incubated on ice for 30–45 min. Cells were washed, suspended in medium containing respective Alexa Fluor 488-conjugated secondary antibodies and then incubated on ice for 30–45 min. Finally, cells were washed, suspended in ice-cold FACS buffer (5% FBS, 1 mM EDTA and 0.02% sodium azide in PBS) and measured the fluorescence intensity using FACS Canto (BD biosciences). Data was analyzed using FlowJo (Tree Star) software and then plotted the mean fluorescence intensity (MFI) as described previously^63^.

### Transcript analysis by quantitative real-time PCR (qRT-PCR) and semiquantiative RT-PCR

Keratinocytes grown in a 35 mm dish was subjected to RNA isolation using GeneJET RNA purification kit (ThermoScientific). The cDNA was prepared from total RNA by using a cDNA synthesis kit (Fermantas). For qRT-PCR, gene transcripts were amplified using the same cDNA for both the gene of interest and the control *18S rRNA* or *GAPDH* in QuantStudio 6 Flex real-time PCR system (Applied Biosystems). The PCR conditions consisted of AmpliTaq Gold activation at 95 °C for 10 min, followed by 40 cycles of denaturation at 95 °C for 20 s, annealing at 58 °C for 25 s and extension at 72 °C for 30 s. A dissociation curve was generated at the end of each cycle to validate the single transcript amplification. The change in SYBR green fluorescence intensity was monitored and then calculated the threshold cycle (C_T_) number. The C_T_ value of the gene was subtracted from respective control to obtain the ∆C_T_ value. The ∆C_T_ value of treated sample was subtracted with the ∆C_T_ value of control to obtain the ∆∆C_T_ value. Finally, the gene expression level relative to the control was expressed as 2^-∆∆CT^, plotted and indicated the fold change. To validate the qRT-PCR data, semiquantative RT-PCR was performed. The gene transcripts were amplified (Bio-Rad S1000 Thermal Cycler) using an equal amount of cDNA from each condition and the gene specific primers (listed in Supplementary Table 2). In all experiments, *GAPDH* or *18S rRNA* was used as loading control. Band intensities were measured, normalized with the loading control, quantified fold change with respect to the control and then indicated in the figure (data not shown for Fig. 2c, f and Supplementary Fig. 1d).

### Nuclear-cytosolic fractionation

Cells in separate dishes were used for cytosolic and nuclear extract preparation and followed protocol as described previously with few modifications^19^. Briefly, cells were washed twice with 1 × PBS before use. For cytosolic extraction, cells in a dish was added with 400 μl buffer (10 mM HEPES-KOH pH 7.5, 3 mM MgCl_2_, 40 mM KCl, 1.0 mM DTT, 0.1 mM PMSF, 0.3% NP-40 and protease inhibitor cocktail) and incubated on ice for 10 min. Cells were scrapped, collected, incubated on ice for additional 15 min, centrifuged at 21,000 × *g* for 30 min and separated the supernatant. Similarly, for nuclear extraction, 500 μl of buffer (10 mM HEPES-KOH pH 7.5, 10 mM KCl, 1.0 mM DTT, 0.1 mM PMSF and protease inhibitor cocktail) was added to the cells in a dish and incubated on ice for 10 min. Further, 0.3% NP-40 was added to the dish and then incubated for 10 min on ice. The cell lysate was collected and centrifuged at 21000 × *g* for 5 min. The obtained nuclear pellet was suspended in 200 μl of buffer (20 mM HEPES-KOH pH 7.5, 400 nM NaCl, 1.0 mM DTT, 0.1 mM PMSF and protease inhibitor cocktail), incubated for 10 min on ice and then centrifuged at 21,000 × *g* for 30 min. Finally, the nuclear lysate was colleced and then probed. Equal protein amounts from cytosolic and nuclear lysates were subjected to immunoblotting after the addition of SDS-PAGE loading dye.

### Immunoblotting

Cell lysates were prepared in RIPA buffer and then subjected to immunoblotting analysis as described previously^63^. Immunoblots were developed with Clarity Western ECL substrate (Bio-Rad) and the luminescence was captured using Image Lab 4.1 software in a Bio-Rad Molecular Imager ChemiDoc XRS+ imaging system, equipped with Supercooled (−30 °C) CCD camera (Bio-Rad). Protein band intensities were measured, normalized with loading control, quantified the fold change with respect to control and then indicated in the figure.

### IFM and image analysis

Cells on coverslips were fixed with 4% formaldehyde (in PBS) and then stained with primary antibodies followed by the respective secondary antibodies as described previously^61^. In some experiments, cells on coverslips were internalized with lysotracker or DQ-BSA, fixed, immunostained and imaged. Bright-field (BF) and immunofluorescence (IF) microscopy of cells was performed on an Olympus IX81 motorized inverted fluorescence microscope equipped with a CoolSNAP HQ2 (Photometrics) CCD camera using 60X (oil) U Plan super apochromat objective. Acquired images were deconvolved and analyzed using cellSens Dimension software (Olympus). The colocalization between two colors was measured by selecting the entire cell excluding the perinuclear area and then estimated the Pearson’s correlation coefficient (*r*) value using cellSens Dimension software. The average *r* value from 10 to 20 cells was calculated and then represented as mean ± s.e.m. Note that the maximum intensity projection of undeconvolved Z-stack images were used for the measurement of *r* values. Analyzed images were assembled using Adobe Photoshop. Half-cell length (in µm, labeled as CL_H_) was measured as the maximum distance between nucleus and the cell periphery using cellSens Dimension software. Likewise, length and width of the nucleus was measured (in µm) by placing the scale bar along the diameter of the nucleus. In Fig. 5, half-cell length (in µm) was measured by masking the BF images with LAMP-1 staining (except bafilomycin A1 condition) to distinguish the cell border. In parallel, the distribution of lysosomes (in µm, labeled as D_L_) in each condition was measured independently from the nucleus to cell periphery (the longest possible distance) using LAMP-1 stained IFM images. Note that the parameter CL_H_ was measured independently in bafilomycin A1 treated cells. Averages of CL_H_ and D_L_ (in µm) in each condition were calculated from approximately 60–80 cells (*n* = 2–3), indicated in Figs. 3, 5 and Table 1 (mean ± s.e.m.), and then plotted using GraphPad Prism software. Using the half-cell length parameter, the percentage of undifferentiated cells (approximately to the maximum size of control cells, < 19 μm) in a given drug treatment/condition was calculated from two or three different experiments and then indicated in Table 1. In Fig. 1d, the percentage of cells showing perinuclear or peripheral distribution of LAMP-1-positive compartments was visually quantified (representing the pattern similar to Fig. 1c) from three different experiments and then plotted. Similarly, number of GFP-LC3 puncta were visually quantified from three different experiments and then plotted. In Supplementary Fig. 2e, cells with altered nuclear morphology (normal, n; structurally altered, SA; notch, NT as indexed) was visually quantified from three different experiments and then plotted.

### Statistical analysis

All statistical analyses were done using GraphPad Prism 5.02 and the significance was estimated by unpaired Student’s *t* test. *p ≤ 0.05; **p ≤ 0.01; ***p ≤ 0.001 and ns, not significant.

## Supplementary information

Supplementary Information

Supplementary Fig. 1

Supplementary Fig. 2

Supplementary Fig. 3

Supplementary Fig. 4

Supplementary Fig. 5
