# Correction: Keratinocyte differentiation promotes ER stress-dependent lysosome biogenesis

**DOI:** 10.1038/s41419-019-1981-7

**Published:** 2019-10-03

**Authors:** Sarmistha Mahanty, Shruthi Shirur Dakappa, Rezwan Shariff, Saloni Patel, Mruthyunjaya Mathapathi Swamy, Amitabha Majumdar, Subba Rao Gangi Setty

**Affiliations:** 1 Department of Microbiology and Cell Biology, Indian Institute of Science, Bangalore, 560012 India; 2 Unilever R&D, Bangalore, 560066 India

**Keywords:** Calcium and vitamin D, Lysosomes, Calcium signalling

**Correction to**: **Cell Death and Disease**

10.1038/s41419-019-1478-4, published online 19 March 2019

Following publication of this article, the authors realized there was an error in Fig. 2b that needed correction. The TFEB panel of Fig. 2b (total lysate) appears to be the same as the TFEB panel of Fig. 2e (cytosolic fraction); the TFE3 panels of Fig. 2b (total lysate) appear to be the same as the TFE3 panels of Fig. 2e (cytosolic fraction) which happened during image assembly. The corrected figure is provided below. This error did not impact the scientific conclusions of the article. We apologize for any inconvenience to the readers.

**Fig. 2:**
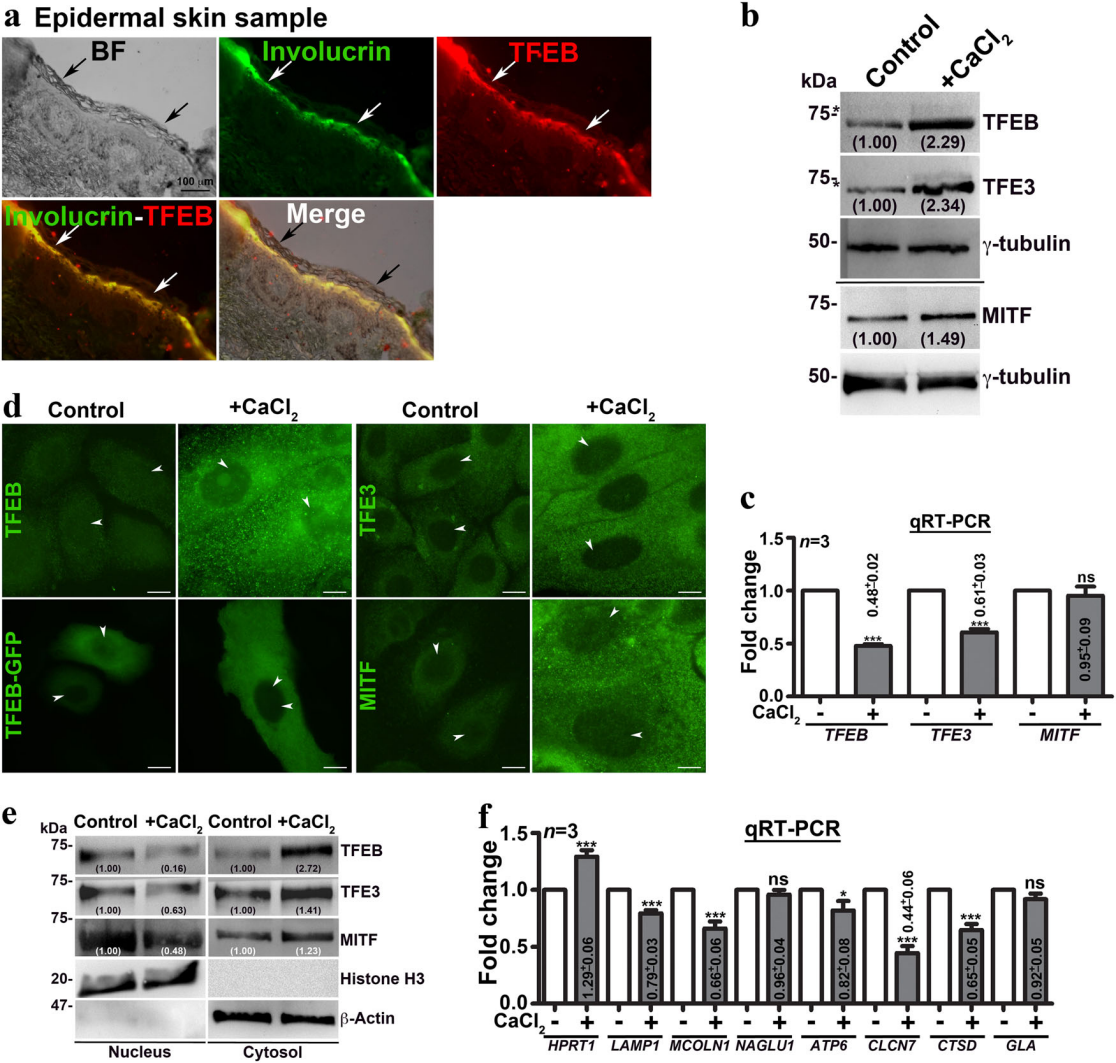
**a** BF and IFM of epidermal skin sample that was immunostained for involucrin and TFEB. Black arrows point to the cornified skin layer and white arrows indicate the involucrin and TFEB-positive layer. Scale bar, 100 µm. **b**, **c** Immunoblotting and qRT-PCR analyses of MiT/TFE TFs (TFEB, TFE3, and MITF). **d**, **e** IFM and nuclear fractionation analyses of keratinocytes for the localization of TFEB-GFP or endogenous MiT/TFE TFs. Arrowheads point to the localization of TFs to the nucleus. Scale bars, 10 µm. In **b** and **e**, the fold change in protein levels is indicated after normalization with respective loading controls. **f** qRT-PCR analysis of various lysosome biogenesis genes. In **c** and **f**, the fold change (mean ± s.e.m.) in gene expression is indicated (*n* = 3). *p ≤ 0.05; ***p ≤ 0.001 and ns, not significant

This has been corrected in both the PDF and HTML versions of the Article.

