# Supplementary material for: Keratinocyte differentiation promotes ER stress-dependent lysosome biogenesis
Source: Cell Death Dis. 2019 Mar 19;10(4):269. doi: 10.1038/s41419-019-1478-4 (PMC6425001; doi:10.1038/s41419-019-1478-4)
Supplement: Supplementary file 1 — Supplementary Information [file 41419_2019_1478_MOESM1_ESM.docx]

**Supplementary Information**

**Keratinocyte differentiation promotes ER stress-dependent lysosome biogenesis**

Mahanty *et al.,*

**Supplementary figure legends**

**Supplementary Fig. 1.**

**CaCl_2_ but not CaCO_3_ induces keratinocyte differentiation and alters nuclear size, but not the endocytic organelles.**

**a** BF and IFM analysis of control, CaCl_2_ or CaCO_3_-incubated cells for 2 or 9 days. Black arrows point to the cell limit and white arrows indicate the distribution of lysosomes. **b** Nuclear length and width (in µm) were measured (~100 nuclei, *n*=3) in each condition and then plotted (mean±s.e.m.). **c** IFM and immunoblotting (IB) analyses of involucrin. Arrow indicates the increased cell size and involucrin expression (both in cytosol and cell surface). The fold change in protein levels is indicated. * indicates non-specific bands detected by the antibodies. **d** qRT-PCR analysis of keratinocyte differentiation markers. The fold change (mean±s.e.m.) in gene expression is indicated (*n*=3). *, *p*≤0.05 and ***, *p*≤0.001. **e** Cells were immunostained for endosomal and lysosomal proteins separately. Arrows indicate the endo/lysosomal protein expression and arrowheads point to LAMP-2-positive lysosomes with respect to Rab9. Nuclei are stained with Hoechst 33258 and the insets are magnified view of the white boxed areas. Scale bars, 10 μm. **f** BF and IFM of epidermal skin sample that was immunostained for LAMP-1. Black arrows point to the cornified skin layer and white arrows indicate the LAMP-1-positive layer. Scale bar, 100 µm.

**Supplementary Fig. 2.**

**Calcium-induced keratinocyte differentiation activates mTOR and autophagy flux and chemical inhibition of mTOR activity enhances differentiation of keratinocytes.**

**a** Immunoblotting analysis of mTOR and autophagy specific genes. Phosphorylation status of few proteins indicated separately. **b-d, f** BF and IFM analyses of keratinocytes for the localization of mTOR, phospho-mTOR, TFEB or LC3 with respective to LAMP-1 or GFP-LC3 alone. Cells were treated with Torin 1 (50 nM) in **d**. Arrowheads point to the LAMP-1-positive organelles with respect to indicated proteins or GFP-LC3-positive autophagosomes (in **f**). Black arrow in **d** shows deformed nucleus. The degree of colocalization (Pearson’s coefficient, *r*) between markers is indicated separately (mean±s.e.m., *n*=3). Nuclei are stained with Hoechst 33258 and the insets are magnified view of the white boxed areas. Scale bars, 10 μm. **e**, **g** The nuclear morphology (as indexed in **e**) or number of autophagosomes (positive for GFP-LC3, **g**) was quantified visually and then plotted (mean±s.e.m., *n*=3). **h** Immunoblotting analysis of autophagy flux in cells as indicated. Cells were treated with or without bafilomycin A1 during the differentiation process. The rate of LC3 conversion (LC3-II/LC3-I) and autophagy flux/turnover (fold change in LC3 conversion with bafilomycin A1) are indicated separately. In **a** and **h**, the fold change in protein levels is indicated. ***, *p*≤0.001 and ns, not significant.

**Supplementary Fig. 3.**

**Keratinocytes alter the expression of involucrin and other differentiation genes upon co-treatment of CaCl_2_ with chemical inhibitors.**

**a, b** IFM analysis of keratinocytes treated with indicated chemical inhibitors (listed in Table 1) alone or in combination with CaCl_2_. Arrows point to the distribution of LAMP-1-positive organelles or expression of involucrin. Note, all images were taken at same exposure as similar to control cells. Scale bars, 10 µm. **c** qRT-PCR analysis of keratinocyte differentiation genes and LAMP-1 at indicated conditions. The fold change (mean±s.e.m.) in gene expression is indicated (*n*=3). Note that keratin 10 levels were unexpectedly increased in only dorsomorphin and CaCl_2_+STO-609 treated conditions. Similarly, bafilomycin treatment showed significant increase in keratin 10 transcript levels. *, *p*≤0.05; **, *p*≤0.01; ***, *p*≤0.001 and ns, not significant.

**Supplementary Fig. 4.**

**Keratinocyte differentiation elevates expression of ER chaperons and activates UPR pathway.**

**a** qRT-PCR analysis of UPR genes (shown in **Fig. 4c**) at indicated conditions. The fold change (mean±s.e.m.) in gene expression is indicated (*n*=3). *, *p*≤0.05; **, *p*≤0.01; ***, *p*≤0.001 and ns, not significant. **b** Immunoblotting analysis of the UPR regulated proteins and their downstream factors. The fold change in protein levels is indicated.

**Supplementary Fig. 5.**

**Differentiation causes fragmentation and dispersal of Golgi and increases colocalization of Golgi tethering proteins with lysosomes in keratinocytes. Golgi function, but not Golgi tethering proteins regulates keratinocyte differentiation and lysosome biogenesis.**

**a-g** IFM analysis of control and differentiated keratinocytes for the localization of ER, transitional ER and Golgi-associated proteins with respect to lysosomes or the organization of Golgi apparatus. In **g**, cells were incubated with fluorescein-conjugated-dextran (0.5 mg/ml) for 6 h, washed and then chased for 12 h. Arrowheads point to the localization of LAMP-1 with respect to other organelles proteins as indicated or GM130 with p230/LAMP-1/fluorescein-dextran. Arrows indicate the distribution of dextran and GM130-colocalized lysosomes. **h,** **i** BF and IFM analyses of cells those were transfected with control or Golgin-97/-245 siRNA followed by an incubation with CaCl_2_ for 48 h. Arrowheads point to the localization of LAMP-1. Arrows indicate the loss in fluorescence staining of Golgi-tethering factors in the respective knockdown cells. **j** IFM analysis of brefeldin A-treated (1 μg/ml for 48 h) control and differentiated keratinocytes for the localization of Golgi-associated protein GM130 with respect to the lysosomes or the organization of Golgi apparatus. Arrowheads point to the localization of GM130 with respect to LAMP-1. Arrows indicate the distribution of GM130. Nuclei are stained with Hoechst 33258 and the insets are magnified view of the white boxed areas. Scale bars, 10 μm.

**Supplementary Table 1.** List of siRNAs used for gene knockdown studies

| **siRNA** | **Sense (5’-3’)**  **Antisense (5’-3’)** | **Position in cDNA** |
| --- | --- | --- |
| Control siRNA  (non-functional) | GAGGACUUGACUAUAGAAGACdTdT  GUCUUCUAUAGUCAAGUCCUCdTdT | Bases 133-155 of  Pallidin (BLOC1S6) (NM_001311255) |
| siGolgin-245 | GAAUGAGGAGCAGGACAUCdTdT  GAUGUCCUGCUCCUCAUUCdTdT | Bases 99-121 of  Golgin-245 (NM_002078) |
| siGolgin-97 | GAUCACAGCCCUGGAACAAdTdT  UUGUUCCAGGGCUGUGAUCdTdT | Bases 769-789 of  Golgin-245 (NM_002077) |

**Supplementary Table 2.** List of primers used for transcript analysis

| **Gene** | **Forward Primer (5’-3’)** | **Reverse primer (5’-3’)** | **Size (bp)** |
| --- | --- | --- | --- |
| *18S rRNA* | TTTCGGAACTGAGGCCATGA | GAACCTCCGACTTTCGTTCTTGA | 155 |
| *ATF4* | CCCGCCCACAGATGTAGTTT | CGCTCGTTAAATCGCTTCCC | 294 |
| *ATF6* | AACAAGACCACAAGACCA | AGGAGGAACTGACGAACT | 151 |
| *ATP6* | CAAGGATGGACGAAGACCCC | AGGGAGAGGAGGCTCAAACT | 211 |
| *BiP* | CGCTTCGAATCGGCGGTACCCAG | TTCTTGTCCTCCTCCTAAGCTTCG | 140 |
| *CHOP* | TTGCCTTTCTCCTTCGGGAC | CAGTCAGCCAAGCCAGAGAA | 178 |
| *CLCN7* | GCTCTGTGATTGTGGCTTTC | TTCAGTGACGTTGACCTTCC | 241 |
| *CTSD* | ACCTCGTTTGACATCCACTATG | AGGATGCCATCGAACTTGG | 191 |
| *GAPDH* | AGTCCACTGGCGTCTTCAC | GCTGATGATCTTGAGGCTGT | 155 |
| *GLA* | CGCTTCATGTGCAACCTTG | TCTGCCTTCTGAATCTCTTTGG | 171 |
| *HPRT1* | AGATGGTCAAGGTCGCAAG | CTGGCGATGTCAATAGGACTC | 245 |
| *Involucrin* | CAACTGGAGCTCCCAGAGCAGC | AACACAGGCTGCTCCAGCTGC | 230 |
| *Keratin 10* | ATGAAATTCAAACCTACCGCAG | CTTGTGGCCTCCGCTGGAGCT | 258 |
| *Keratin 18* | AGTCCTTGGAGATCGACCTGGA | GGTAGGTGGCGATCTCAGCCT | 370 |
| *LAMP1* | TTCAAGGTGGAAGGTGGC | CCAGAGAAAGGAACAGAGGC | 222 |
| *Loricrin* | TCTGGCTGCGGCGGAGGCTCCT | TCCAGAGGAACCACCTCCGCAG | 230 |
| *MCOLN1* | CCACATCCAGGAGTGTAAGC | CCAGCCATTGACAAATTCCAG | 226 |
| *MITF* | TCAGACACCAGCCATAAACG | TCCATCAAGCCCAAGATTTCC | 446 |
| *NAGLU* | CTTTCAATGAGATGCAGCCAC | TCAGCAAACAGGTCCAGAAC | 220 |
| *PERK* | GCAGACGGTTCTGACCCCAATG | TGATGTTTATAGCTTCAGGTCG | 311 |
| *TFE3* | GCAGGCGATTCAACATTAACG | AAAGTGCAGGTCCAGAAGG | 459 |
| *TFEB* | AGTACCTGTCCGAGACCTATG | TGATGTTGAACCTTCGTCTCC | 474 |
| *XBP1* | TTACGAGAGAAAACTCATGGCC | GGGTCCAAGTTGTCCAGAATGC | 257 |
