# Supplementary figures and images for: Keratinocyte differentiation promotes ER stress-dependent lysosome biogenesis
Source: Cell Death Dis. 2019 Mar 19;10(4):269. doi: 10.1038/s41419-019-1478-4 (PMC6425001; doi:10.1038/s41419-019-1478-4)

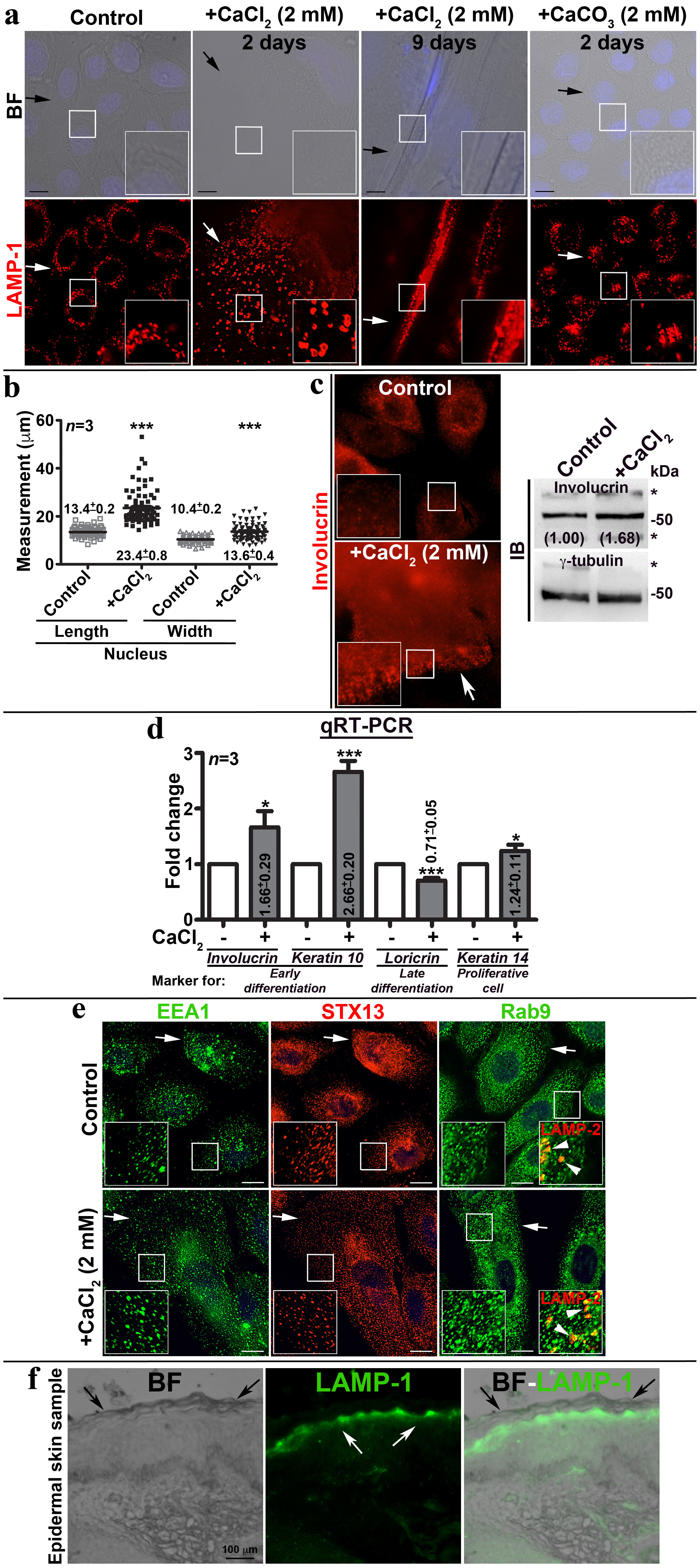

Supplement: Supplementary file 2 — Supplementary Fig. 1 [file 41419_2019_1478_MOESM2_ESM.jpg]

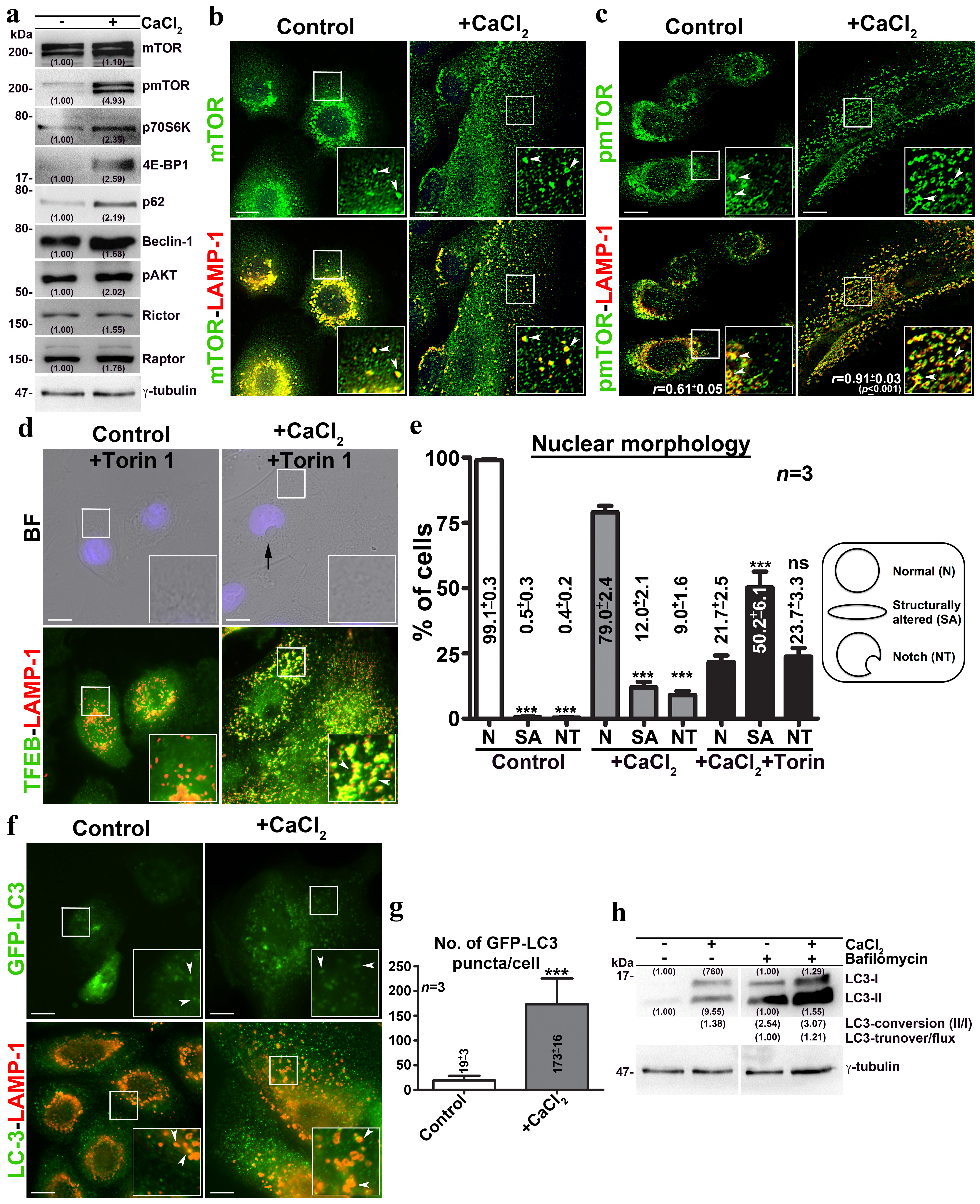

Supplement: Supplementary file 3 — Supplementary Fig. 2 [file 41419_2019_1478_MOESM3_ESM.jpg]

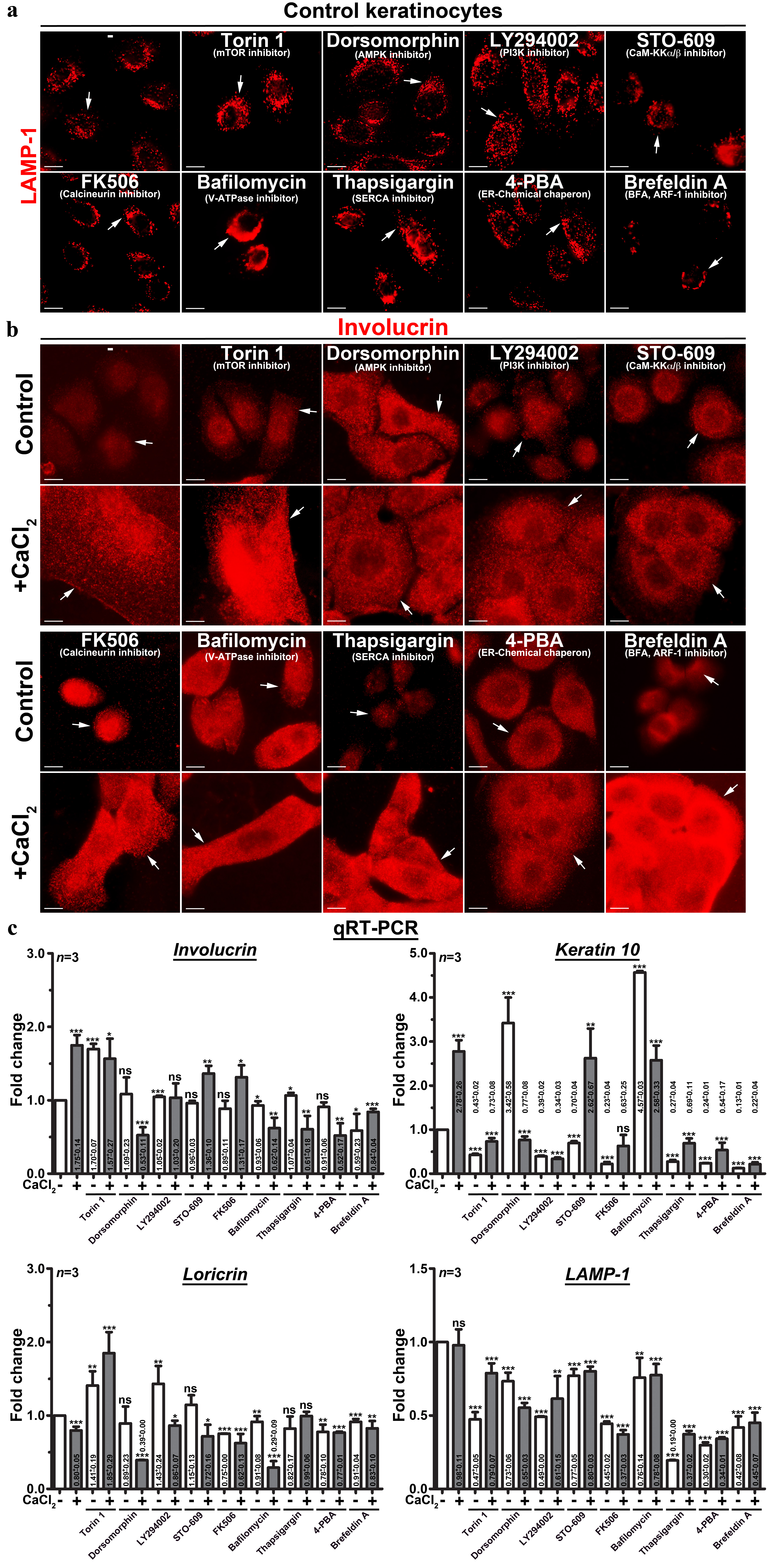

Supplement: Supplementary file 4 — Supplementary Fig. 3 [file 41419_2019_1478_MOESM4_ESM.jpg]

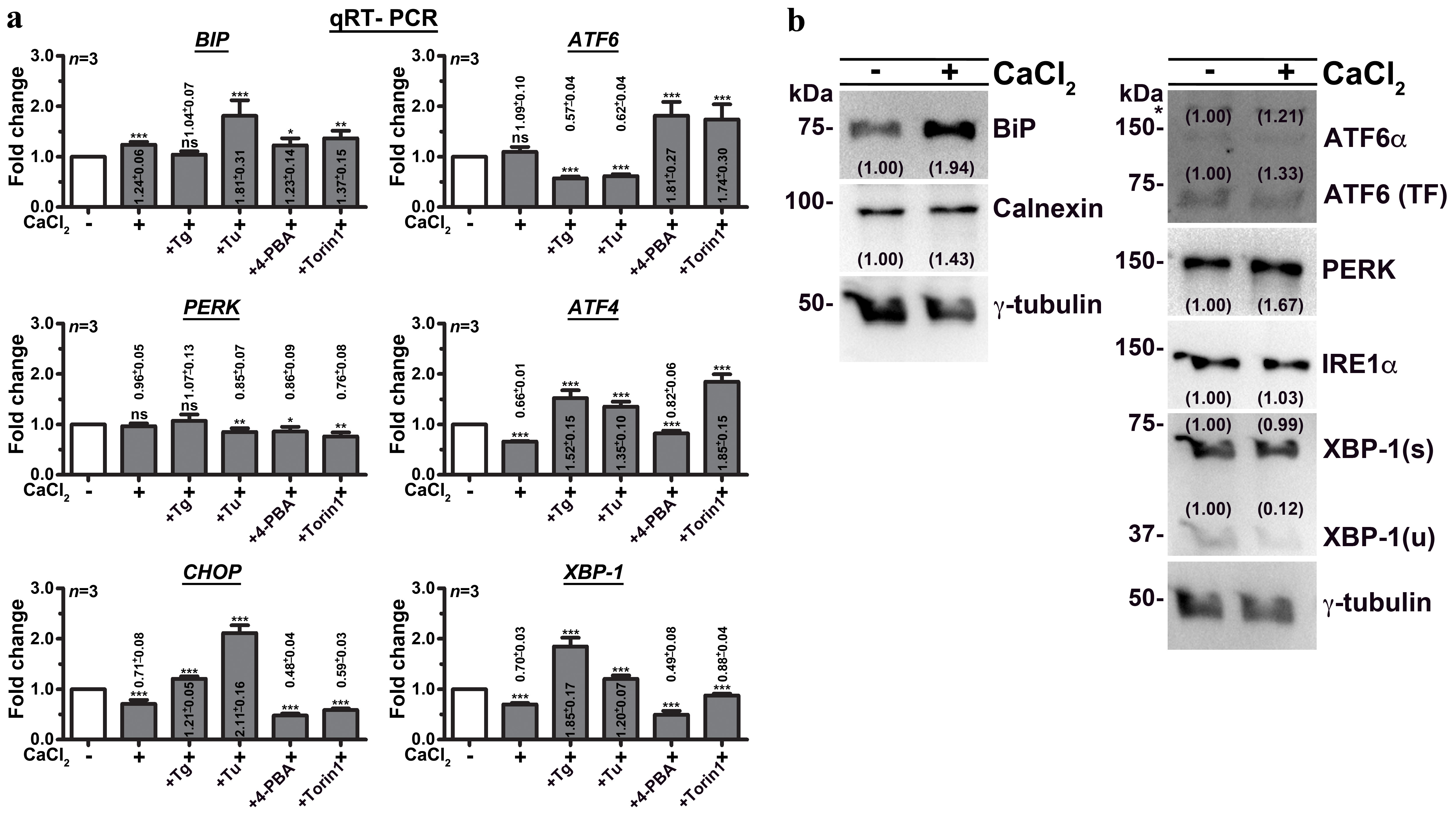

Supplement: Supplementary file 5 — Supplementary Fig. 4 [file 41419_2019_1478_MOESM5_ESM.jpg]

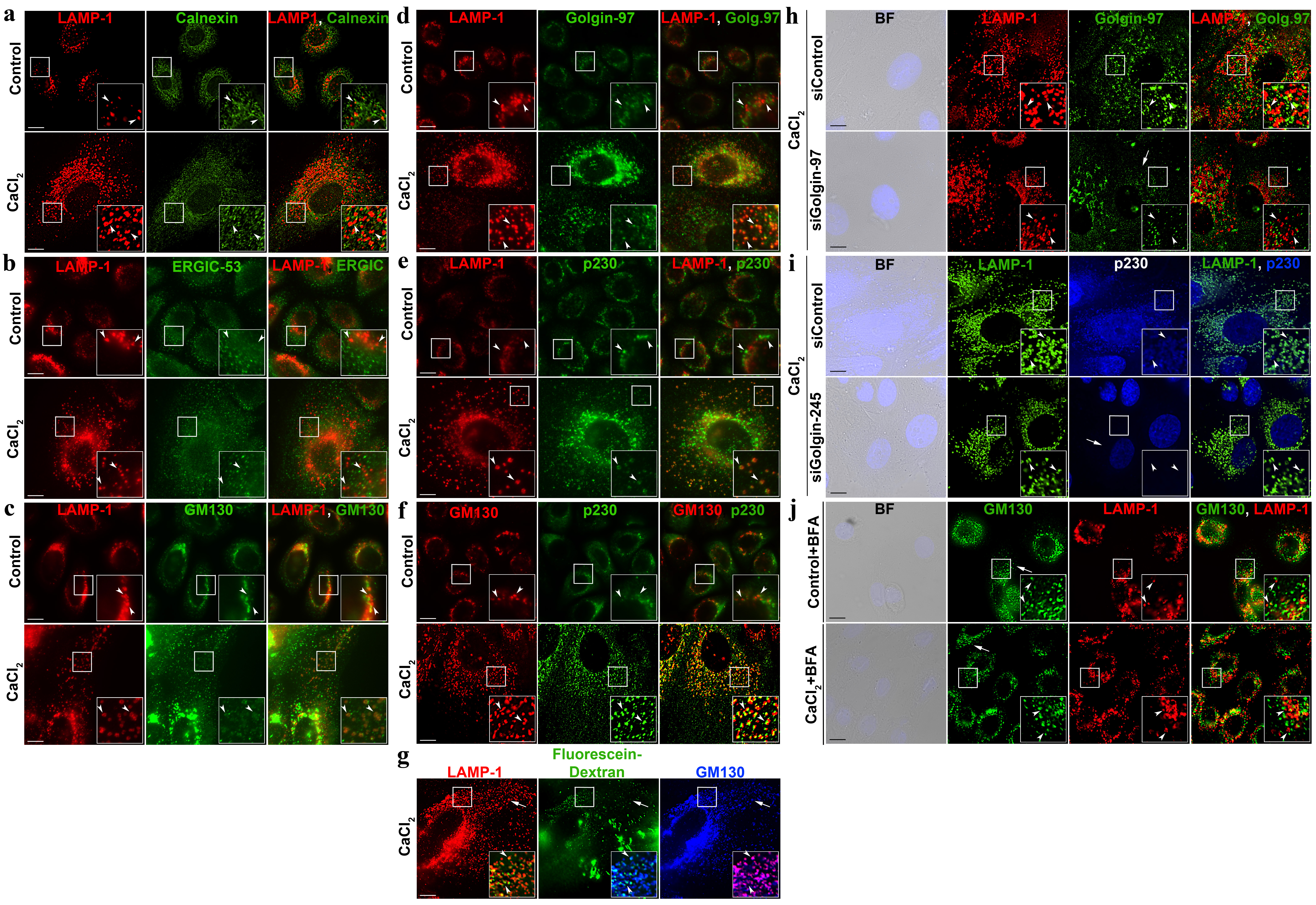

Supplement: Supplementary file 6 — Supplementary Fig. 5 [file 41419_2019_1478_MOESM6_ESM.jpg]
